# Attachment Concepts and Suicidal Thoughts and Behaviours in Adolescents: A Systematic Review and Meta‐Analysis

**DOI:** 10.1002/cpp.70251

**Published:** 2026-03-17

**Authors:** Xingyu Wang, Daniel Pratt, Qinyi Zhong, Katherine Berry

**Affiliations:** ^1^ Division of Psychology and Mental Health, School of Health Sciences, Faculty of Biology, Medicine & Health University of Manchester Manchester UK; ^2^ Department of Research and Innovation Greater Manchester Mental Health Trust Prestwich UK

**Keywords:** adolescent, attachment, meta‐analysis, suicidal ideation, suicide attempt

## Abstract

This review provided a meta‐analysis, narrative synthesis and quality appraisal of quantitative studies examining associations between adolescent attachment concepts and suicidal ideation and suicide attempts among adolescents. A systematic search of PsycINFO, PubMed, EMBASE, Web of Science and CINAHL was undertaken. Studies on attachment and suicidal ideation or attempts in adolescents aged 10–24 years were included. Fifty‐four studies met inclusion criteria. Subgroup meta‐analyses showed small associations with suicidal ideation for attachment security (*r* = −0.161) and attachment anxiety (*r* = 0.198). Avoidance was not significantly associated with ideation (*r* = 0.061). Narrative synthesis suggested a weak link between attachment anxiety and suicide attempts, while the association with avoidance remained uncertain. Lower attachment quality, lower parental care and higher parental overprotection were associated with both ideation and attempts. Heterogeneity was high across most subgroups, and potential publication bias was detected for the parental overprotection group, warranting caution in interpretation. Findings also suggested mediating roles for interpersonal and emotional difficulties and moderating roles for social support and environmental sensitivity. These findings align with adult models of suicidal thoughts and behaviour, suggesting they can be extended to adolescents. Based on these findings, clinical implications could include enhancing attachment security, improving emotional management, addressing difficulties in developing relationships with others, providing support for adolescents experiencing low social support and fostering positive family and school environments. Future research should evaluate these interventions using randomised controlled trials with sufficient samples to examine mediators and moderators of change.

## Summary

- Insecure attachment is related to both suicidal ideation and suicide attempts in adolescents.
- Emotional problems and interpersonal factors serve as mediators, while social support and environmental sensitivity function as moderators in these relationships.
- Randomised controlled trials with sufficient sample sizes are needed to test interventions that target mediators and moderators to inform clinical practice.

## 1 Introduction

Suicide is the second leading cause of death among young people aged 10–24 years worldwide (Hawton et al. 2012). In addition, adolescent suicide may be substantially underreported due to coroners' reluctance to assign a suicide verdict to young people (Gosney and Hawton 2007). Suicidal thoughts and behaviours (STB), including suicidal ideation (SI) and suicide attempts (SA), are significant risk factors for death by suicide (Cybulski et al. 2022; Bridge et al. 2006). Improving understanding of SI and SA among adolescents is crucial for developing more refined suicide models that can inform the development of more targeted interventions to prevent suicide. Adolescent STB results from complex genetic, biological, psychological, social and cultural interactions (Hawton et al. 2012). Most theoretical models emphasise a diathesis‐stress explanation of STB (Mann et al. 1999; O'Connor and Kirtley 2018). Early vulnerabilities resulting from genetic factors or early trauma experiences lead to emotion dysregulation (Dvir et al. 2014), limited problem‐solving abilities (Malhi et al. 2019) and increased sensitivity to defeat (Hammen 2005), increasing stress susceptibility. Therefore, when facing later life stress, these vulnerabilities can overwhelm coping mechanisms, thus increasing the risk of STB (Van Heeringen 2012). These models also consistently highlight the crucial role of interpersonal factors in developing and maintaining STB (O'Connor and Kirtley 2018; Joiner 2005).

Attachment theory is a key framework for understanding early vulnerability and interpersonal problems related to STB (Venta and Sharp 2014). Theoretical models and previous studies have also suggested the associations between insecure attachment and STB (Adam 1994; Sheftall et al. 2014). Attachment is a lifelong process influencing the ability to form and maintain intimate bonds (Bowlby 1969). Early repeated attachment experiences with attachment figures (typically caregivers in early life) establish mental representations of the self and others called Internal Working Model (IWM), which guide emotion regulation and social information processing (Bowlby 1969).

There are individual differences in IWMs based on the nature of early interactions between the infant and their caregivers, which are referred to as attachment styles: secure, anxious and avoidant. Secure attachment results from consistent, responsive and sensitive caregiving (Shaver and Mikulincer 2002). It is associated with positive self‐ and other‐perceptions, the capacity to build intimate relationships and effective distress management (Shaver and Mikulincer 2021). Anxious attachment is associated with inconsistently available and only occasionally responsive caregiving. It is marked by hyperactivating strategies (e.g., amplifying distress to attract attention), low self‐worth, being overwhelmed by negative affect and hypersensitivity to rejection (Mikulincer and Shaver 2019). Avoidant attachment is associated with consistently unavailable caregiving. It is linked to deactivating strategies (e.g., avoiding proximity and suppressing emotions) (Mikulincer and Shaver 2010) and is characterised by negative expectations of others' availability and responsiveness and difficulty forming close relationships (Mikulincer and Shaver 2012). Disorganised attachment is an additional pattern identified to describe infants who do not fit existing styles (Main 1996). It arises when caregivers are seen as a source of fear or threat, such as in cases of abuse (Main and Solomon 1986; Main and Hesse 1990; Adam et al. 2013). Additionally, caregivers with trauma or loss may perceive the child as a threat and behave frighteningly, leading to disorganised attachment in the infant (Main and Hesse 1990; Schechter et al. 2004; Iyengar et al. 2014). Infants with disorganised attachment lack a cohesive way to meet their attachment needs, resulting in difficulties with emotion regulation (Main and Solomon 1986; Main et al. 1985). During adolescence, attachment relationships broaden to include peers as attachment figures, and these relationships are also shaped by adolescents' IWMs (Gorrese and Ruggieri 2012; Delgado et al. 2022).

**FIGURE 1:**
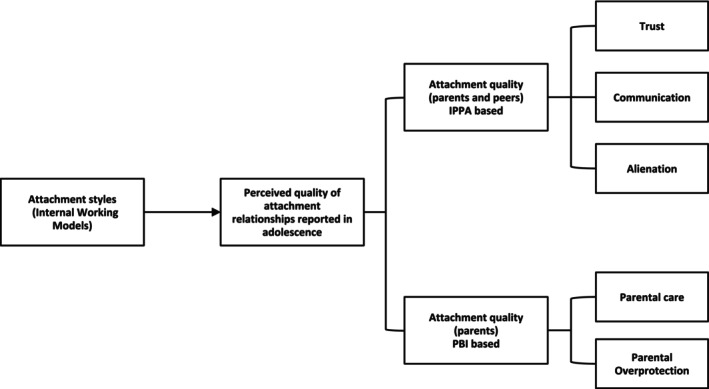
Conceptual framework linking attachment styles and attachment‐related concepts in adolescence.

Research on attachment processes within adolescence often employs attachment‐related concepts to assess adolescents' cognitive representations of their attachment relationships with parents and peers during this developmental stage. Measures widely used with adolescents include the Inventory of Parent and Peer Attachment (IPPA) and the Parental Bonding Instrument (PBI). These measures capture adolescents' attachment‐relevant perceptions of relationship quality with attachment figures. These perceptions may be influenced by adolescents' IWMs underlying adolescents' attachment styles (see Figure 1 for a conceptual illustration) (Delgado et al. 2022; Parker et al. 1979; Vivona 2000). The IPPA assesses attachment through three key domains: trust, communication and alienation. Overall attachment quality is determined by the combined scores of these three dimensions. These attachment‐related domains assess perception of attachment figures' availability, sensitivity and responsiveness (Armsden and Greenberg 1987). High trust and communication combined with low alienation are related to greater attachment security, whereas the reverse pattern is related to lower attachment security (Armsden and Greenberg 1987). The PBI measures attachment through parental care and overprotection (Parker et al. 1979). Parental care reflects adolescents' perceptions of caregivers' availability during distress, and parental overprotection reflects perceptions of how caregivers support healthy exploration of the environment. Higher care together with lower overprotection is associated with greater attachment security, whereas the reverse pattern is associated with lower attachment security (Parker et al. 1979). Taken together, IPPA and PBI constructs, as well as attachment‐related concepts assessed using other measures, primarily capture adolescents' perceived relationship quality and caregiving experiences with attachment figures. On the IPPA, lower trust and communication and higher alienation indicate lower perceived security in the attachment relationship. On the PBI, lower parental care and higher overprotection similarly reflect negative bonding experiences, which are linked to lower attachment security. Overall, more positive attachment‐related concepts are associated with greater attachment security, whereas more negative concepts are associated with insecure attachment patterns, such as higher attachment anxiety, avoidance or disorganisation.

Adam's developmental model of suicide, which draws on attachment theory, proposes that early attachment experiences shape later risk of STB by influencing the IWMs (Adam 1994). Negative attachment experiences may contribute to IWMs characterised by negative views of the self and others, which are linked vulnerabilities such as low self‐worth, poor emotional regulation and relationship difficulties. These vulnerabilities can increase anxiety, anger, hopelessness and the risk of suicidal behaviours in response to stressors like loss, rejection or disappointment (Adam et al. 1996). Although Adam's model does not specify particular attachment styles or attachment‐related concepts, it can be applied to explain the association between attachment styles and related concepts and STB. Secure attachment, reflecting IWMs characterised by positive views of the self and others, supports the development of emotion regulation capacities, and expectations that support from others will be available. Therefore, adolescents with secure attachment are able to manage distress and seek support from close relationships during difficult periods, reducing the risk of suicidal behaviours (Adam 1994). Conversely, insecure attachment styles (i.e., avoidant, anxious and disorganised attachment) reflect IWMs characterised by negative views of the self and/or others. These patterns may be associated with greater difficulties in emotion regulation and less confidence in receiving support, increasing the risk of STB (Adam 1994). The IWMs guide adolescents' information processing in later relationships, shaping how adolescents perceive the quality of relationships with parents and peers (Bowlby 1969). IWMs characterised by negative views of others are associated with lower trust and communication and higher alienation, as captured by the IPPA, and with lower parental care and higher parental overprotection, as captured by the PBI (Delgado et al. 2022; Parker et al. 1979; Vivona 2000). These negative representations lead adolescents to expect parents or peers to be unavailable or unresponsive when reaching out for support (Collins and Feeney 2004). This expectation may reduce help‐seeking and limit access to support, which may be related to risk of STB (Adam 1994).

Previous reviews examining adult or mixed‐age samples have identified associations between insecure attachment and STB (Green et al. 2020; Miniati et al. 2017; Zortea et al. 2021; Macneil et al. 2023). However, few have focused specifically on adolescents. Adolescence is a period marked by biological and social role transition difficulties and by relationship challenges, including navigating peer relationships and adjusting relationships with parents (Dallos 2023; Steinberg 2017). These transition and relationship difficulties heighten emotional distress and may strengthen the association between attachment and STB (Dallos 2023; Steinberg 2017; Xu et al. 2022). One meta‐analysis including developmental stage (adolescents vs. adults) as a moderator found no significant association between insecure attachment and STB in adolescents (Macneil et al. 2023). These findings contrasted with systematic reviews of adolescents that reported significant associations (Woo et al. 2022; Yang et al. 2023). Woo et al. conducted a systematic review and found evidence that insecure attachment was related to both SA and nonsuicidal self‐injury (NSSI) in children and adolescents under 18 (Woo et al. 2022). Nearly half of the included studies in this review were longitudinal and assessed attachment to both parents and peers. However, no meta‐analysis was conducted due to substantial heterogeneity in samples and measures. Yang et al. later conducted a systematic review and meta‐analysis on the relationship between attachment to parents and SI among adolescents (mean age 12.60–15.99 years) and young adults (mean age 18.70–26.00 years) (Yang et al. 2023). They found low‐quality parental attachment significantly associated with increased SI in both age groups. This review considered a broader age range compared to Woo et al.'s review, aligning with evolving definitions of adolescence (Sawyer et al. 2018). There is now a consensus that adolescence extends into the mid‐20s, reflecting delayed transitions to adult roles and continued brain development during this period (Sawyer et al. 2018). Despite its strengths, Yang et al.'s review had limitations. During adolescence, attachment networks broaden to include peers, who become particularly important attachment figures at this stage (Gorrese and Ruggieri 2012; Delgado et al. 2022). However, this review focused only on parental attachment, overlooking peer attachment. In addition, it treated insecure attachment as a global construct (attachment quality), without distinguishing between specific attachment styles or attachment‐related concepts.

Furthermore, neither of these previous reviews explored factors mediating or moderating the relationship between insecure attachment and STB in adolescents. A systematic review focused upon adults explored the relationship between attachment and STB, suggesting different types of insecure attachment may lead to STB through distinct pathways (Zortea et al. 2021). Specifically, individuals with anxious attachment may use STB to seek help and express distress, while those with avoidant attachment may do so to cope with adversity or emotional pain due to limited support‐seeking opportunities. Given the unique psychological and social development in adolescence, findings based on adults may not be appropriate to generalise (Sisk and Gee 2022; Crone and Dahl 2012; Jaworska and MacQueen 2015). Understanding the mediators in adolescents would identify plausible mechanisms linking different attachment styles to STB, thereby informing intervention targets for adolescents with varying attachment styles and supporting more targeted strategies that could more effectively reduce adolescents' STB risk. Regarding moderators, the aforementioned adult review examined gender differences but found the evidence unclear due to insufficient and inconsistent data (Zortea et al. 2021). Identifying adolescent‐specific moderators would clarify for whom and under what circumstances attachment‐related STB risk is heightened across attachment styles, supporting the identification of higher risk subgroups and strengthening early prevention efforts.

This systematic review and meta‐analysis aimed to address gaps in existing reviews. First, it focused specifically on adolescents aged 10 to 24 years, reflecting evolving definitions of adolescence due to social and developmental changes (Sawyer et al. 2018). Second, this review included a broad range of attachment figures and concepts. Beyond attachment to parents, it considered attachment to peers, family members and general networks. This review also incorporated both traditional attachment styles (secure, anxious, avoidant and disorganised) and attachment‐related concepts captured by attachment‐specific measures, such as the IPPA and PBI. The attachment‐related concepts measured by the IPPA and PBI were included in this review as part of the broader attachment framework and treated as complementary indicators. In the following sections, both traditional attachment styles and these attachment‐related constructs are referred to collectively as attachment concepts. Finally, this review examined potential moderators and mediators affecting attachment concepts' relationships with SI or SA. By addressing these gaps, it aims to improve understanding and inform adolescent suicide prevention.

## 2 Aim and Objectives

This review aims to examine the relationship between attachment concepts and SI and SA in adolescents. Attachment concepts are broadly defined to include the following: (1) traditional attachment styles (secure, avoidant, anxious and disorganised); (2) IPPA dimensions (trust, communication, alienation and attachment quality); (3) parental bonding dimensions (care and overprotection); and (4) other attachment‐related constructs. The specific aims are outlined below.

1. Appraise the quality of the available evidence.
2. Investigate the relationship between attachment concepts and SI.
3. Investigate the relationship between attachment concepts and SA.
4. Identify factors that mediate or moderate these relationships.
5. Make recommendations for future studies and suicide prevention interventions.

## 3 Method

This systematic review described its findings in accordance with the Preferred Reporting Items for Systematic Review and Meta‐analysis (PRISMA) (Moher et al. 2010). The review protocol was registered in advance on PROSPERO (Registration number: CRD42023424800).

### 3.1 Search Strategy

Subsequent to the running of trial searches to test and refine search terms and to check that key papers were retrieved, five databases (PsycINFO, PubMed, EMBASE, Web of Science and CINAHL) were systematically searched to identify studies for inclusion. Several domain‐specific and related search terms were combined with Medical Subject Headings (MeSH) and subject headings to broaden the search and identify relevant studies. The search combined terms for attachment and close relationships, STB and self‐harm and adolescents and young people. A full Boolean search string example is provided in Appendix S1. Searches were completed in February 2023 and repeated in May 2025.

### 3.2 Eligibility Criteria

Eligible studies that were published in peer‐reviewed journals and written in English or Chinese (the first author's first language) were included. They reported quantitative associations between attachment concepts and SI alone, SA alone or both (analysed separately). Participants were adolescents aged 10–24 years. Detailed inclusion and exclusion criteria are shown in Table 1.

**TABLE 1:** Inclusion and exclusion criteria.

	Inclusion criteria	Exclusion criteria
Age range	Adolescents aged 10 to 24 years old	Participants who were under 10 years or over 24 years of age
Publication type	Published in a peer‐reviewed journal	Not published in peer‐reviewed journals, as they had not undergone a rigorous peer‐review process (e.g., conference reports and dissertations)
Language	Written in English or Chinese (as it is the first author's first language)	Not written in English or Chinese language
Study design	Observational studies using prospective cohort, cross‐sectional or case–control designs to examine the relationship between attachment concepts and suicidal thoughts and behaviours;	Editorials, review papers and commentaries
	Intervention studies or controlled trials reporting on the association between attachment concepts and suicidal thoughts and behaviours	Qualitative studies and case studies
Attachment‐related criteria	Included a self‐report, interview or observational measure of attachment concepts	Assessed constructs related to, but distinct from, attachment concepts, such as school attachment, connections to parents and friends or family functioning
Suicidal outcomes' criteria	Included a measure (single question, self‐report questionnaire or interview‐based assessment) assessing suicidal ideation alone, suicide attempts alone or both separately	Examined only self‐harm without suicidal ideation (e.g., nonsuicidal self‐injury) or self‐harm where the intent to end life was unknown or uncertain
		Grouped individuals with suicidal ideation and/or suicide attempts into a combined category. This review aimed to distinguish attachment concepts differences between individuals with suicidal ideation and those who have attempted suicide, which a combined category does not allow

### 3.3 Study Screening and Selection

After removing duplicates, the first author independently screened all titles and abstracts to exclude ineligible studies, followed by a full‐text review of the remaining papers to identify those meeting the inclusion criteria. A subset of the studies was then independently screened to assess interrater reliability. Estimates of interrater reliability revealed acceptable levels of agreement (McHugh 2012) at title and abstract (kappa = 0.749, *p* < 0.001) and full‐text stages (kappa = 0.888, *p* < 0.001). The slightly lower agreement at the title and abstract screening stage likely reflects the more interpretive nature of this stage, where inclusion decisions are often based on limited information (McHugh 2012; O'Connor et al. 2019). Disagreements were resolved through discussion with the wider research team. To ensure comprehensive coverage and avoid missing relevant studies, the backward search was conducted by examining references of included studies and two existing reviews (Woo et al. 2022; Yang et al. 2023). The forward search using Web of Science identified studies citing the included papers. All identified studies were screened according to the eligibility criteria.

### 3.4 Data Extraction

Key study characteristics (e.g., author, year, country, sample size, age range, attachment and suicide measures) were extracted using a standardised data extraction form. Due to the fact that measures vary between studies, Appendix S3 maps each measure to its attachment construct and whether it assessed SI or SA. The first author extracted data using a bespoke form. A postgraduate research student involved in screening checked the extraction, with disagreements resolved through discussion and consultation with the wider team. The first author contacted study authors for missing methodological details (e.g., age range), statistical data for effect sizes and full texts. Regarding attachment‐related concepts, all included studies measured these constructs as dimensional, using continuous scores. Regarding attachment styles, the majority of studies treated attachment as dimensional, using continuous scores for secure, anxious, avoidant and disorganised attachment in the analyses, rather than assigning participants to a single attachment category. Only one study used a categorical classification by identifying each participant's predominant attachment style based on the highest of the four dimensional ratings (Venta and Sharp 2014). Therefore, except for this study, attachment styles and attachment‐related concepts in the present review were coded as dimensional variables, and the results are interpreted as reflecting dimensional variation rather than categorical differences.

### 3.5 Quality Assessment

Study quality was assessed by the Quality Assessment Tool for Observational Cohort and Cross Sectional (National Heart, Lung, and Blood Institute (NHLBI), and National Institutes of Health (NIH), n.d.). This tool has been used in previous reviews on STB in young people, with established validity and reliability (Abdelraheem et al. 2019; Perquier et al. 2021). Included studies were evaluated on six criteria: (1) research question; (2) study population and recruitment; (3) measures and measurement process; (4) blinding of outcome assessors; (5) follow‐up rate; and (6) statistical analyses. Assessment of each question was categorised as ‘yes’, ‘no’, ‘cannot determine’, ‘not applicable’ and ‘not reported’. The ratings on the different questions informed the overall quality categorisation of ‘good’, ‘fair’ or ‘poor’. Detailed information for ratings is provided in Appendix S4. The first author and a postgraduate research student independently assessed all studies. Interrater reliability was excellent across all items and overall ratings (ICC = 0.986, *p* < 0.001). Disagreements were resolved through discussion with the wider research team.

### 3.6 Analysis

#### 3.6.1 Meta‐Analysis

Several subgroup analyses were conducted to explore the relationships between specific attachment concepts and SI or SA. Comprehensive Meta‐Analysis 3.0 was used to calculate effect sizes. Pearson's r correlation coefficient was used as the primary effect size metric, given its frequent use across included studies. Other statistics (e.g., mean differences) were converted to Pearson's r if conceptually consistent with this effect size family (Borenstein et al. 2021). Studies that did not provide the required data to allow this conversion were not included in the meta‐analysis. The random effects model was applied for pooling the effect size to account for heterogeneity (Viechtbauer 2007). To ensure sufficient power, subgroup analyses were only conducted when at least five studies were available (Jackson and Turner 2017). Subgroups with fewer studies were included in the narrative synthesis. To ensure consistency and minimise bias across studies, specific data handling procedures were applied (see Appendix S2). Correlation effect sizes were interpreted using Cohen's guidelines: *r* = 0.10–0.29 (small), 0.30–0.49 (medium) and 0.50 and above (large) (Cohen 2013).

Cochran's *Q*‐test and *I* ^2^ statistic (25% = low; 50% = moderate, 75% = high heterogeneity) (Higgins et al. 2003) were employed to evaluate the heterogeneity. Publication bias was assessed using Egger's test (Egger et al. 1997), visual inspection of funnel plots and the ‘Trim‐and‐Fill’ method (Duval and Tweedie 2000). ‘One study removed’ sensitivity analyses were conducted to assess whether any individual study substantially influenced the overall results. This method also examined whether study quality affected the meta‐analysis findings.

#### 3.6.2 Narrative Synthesis

Studies were categorised into four domains based on attachment classification: (1) secure, avoidant, anxious and disorganised attachment; (2) trust, communication, alienation and attachment quality (combination of the trust, communication and alienation); (3) care and overprotection; and (4) other attachment concepts. Within each domain, relationships between attachment and SI or SA were first described narratively. Where data allowed, effect sizes were reported or converted to Pearson's *r* to enhance comparability across studies. Findings were then compared within each domain, considering study design, population and quality to explain inconsistencies. Similarities and differences in findings were also explored between the narrative synthesis and the meta‐analysis.

Mediator findings were organised following Adam's model of suicide, which highlights intrapersonal factors (e.g., emotional states and self‐worth) and interpersonal difficulties. These findings were summarised and compared within each group, with possible explanations for inconsistencies explored. Moderator findings were synthesised, though few studies examined potential moderators. Throughout the narrative synthesis, the findings from the quality appraisal were used to help interpret the weight of findings.

## 4 Results

### 4.1 Overview of the Included Studies

**FIGURE 2:**
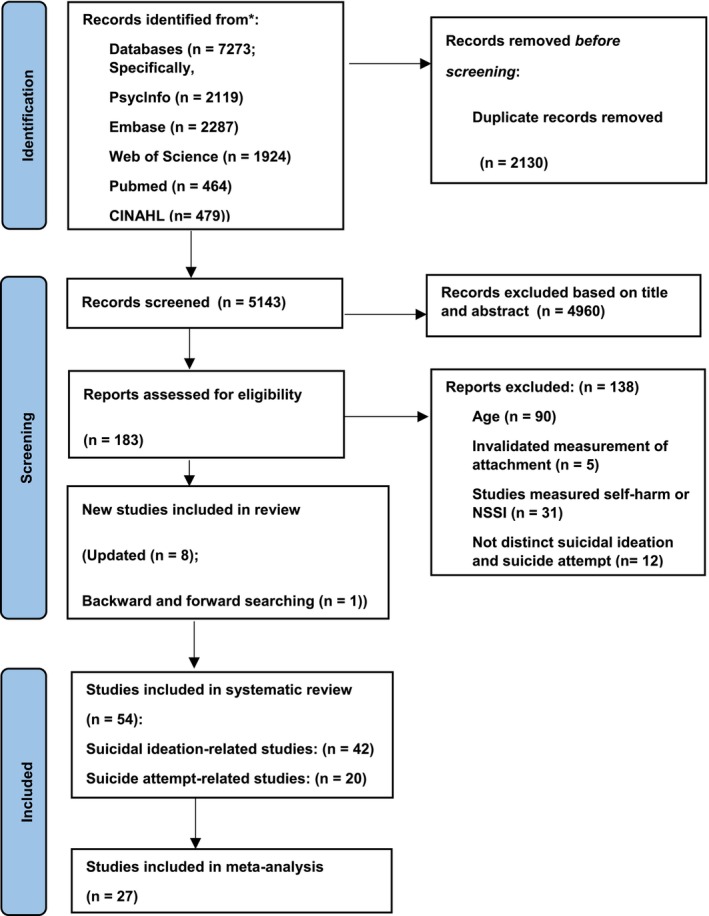
PRISMA flow chart.

Figure 2 displays the screening procedure, including the number of included and excluded studies, as well as the reasons for exclusion at each phase. During the initial search on 15 February 2023, a total of 7273 studies were identified and 2130 were removed as duplicates. From the 5143 remaining studies, 4960 studies were excluded based on the screening of titles and abstracts, and 183 studies were evaluated for eligibility at the full‐text level. Forty‐five of these studies were deemed to meet inclusion criteria. An updated search conducted on 19 May 2025 identified 888 additional studies. Of these, 50 were examined for eligibility at the full‐text level, and a further eight studies were identified as meeting inclusion criteria. One additional study was identified through backward and forward searching. Thus, our systematic review includes a total of 54 papers, with 27 studies reporting sufficient data to enable inclusion within the meta‐analysis.

### 4.2 Study Characteristics

The characteristics of the included studies are displayed in Table 2. The total number of participants was 83,356. Regarding study design, nine studies analysed data from longitudinal studies, seven from randomised controlled trials (RCT), 14 studies were between‐group cross‐sectional design, and 22 studies applied correlational cross‐sectional design. Across all studies, the female to male ratio was 1.07:1 (excluding one study without sex/gender data), and the mean age was 14.84 years (six studies gave only age ranges). Thirty‐three studies comprised non‐clinical samples only (*n* = 79,398), 19 clinical only (*n* = 3078) and the remaining two studies both. Twenty‐two studies were conducted in North America (*n* = 29,798), 14 studies in Asia (*n* = 40,183), 14 studies in Europe (*n* = 10,884), two studies in Oceania (*n* = 1946), one study in South America (*n* = 395) and one study in Africa (*n* = 150).

**TABLE 2:** Study characteristic of included papers.

Author	Study design	Country	Sample size	Age	Clinical or non‐clinical	Measurement of attachment	Attachment concept	Attachment relationship	Measurement of suicidal thoughts and behaviours	Type of suicidal thoughts and behaviours
Bakken et al. 2025	Longitudinal study (1 year)	Norway	2464	12.5–15.7 (13.7 ± 0.6)	Non‐clinical	IPPA	Total score	Mother, father and peer	MFQ‐SI (5); single‐item measure‐SA	SI, SA
Dong et al. 2024	Longitudinal study (4 years)	China	7010	15 (15.47 ± 0.78)	Non‐clinical	Modified version of PBI	Care	Parent	PANSI (translated version)	SI
Shin and Bae 2024	Longitudinal study (3 years)	Korea	6773	14 (14)	Non‐clinical	IPPA	Alienation	Parent	Single‐item measure	SI
Bakken et al. 2024	Data from longitudinal study (13 years)	Norway	2423	14–15 (14.9 ± 0.6)	Non‐clinical	IPPA, IPPA‐9 (peer) (translated version)	Total score	Mother, father and peer	MFQ‐SI (5) (translated version)	SI
Yang et al. 2023	Correlational study	China	479	12–15 (14.29 ± 0.81)	Non‐clinical	IPPA‐20 (translated version)	Total score	Parent	SSOSI	SI
Myerson et al. 2023	Between‐groups study	The United States	690	12–17 (15.13 ± 1.50)	Clinical	ECR‐RS	Secure, preoccupied, dismissing and fearful	Mother and father	Single‐item measure	SA
Cohen and Stutts 2023	Correlational study	The United States	9900	13–18	Non‐clinical	Modified version of PBI	Care, control	Parent	Single‐item measure	SI, SA
Guo et al. 2023	Longitudinal study (1 year)	China	4171	10–19 (14.99 ± 1.52)	Non‐clinical	IPPA (translated version)	Trust, communication, alienation	Mother, father and peer	PANSI (translated version)	SI
Lara Leben Novak et al. 2023	Correlational study	Slovenia	217	11–18 (15.8 ± 1.5)	Clinical	ECR‐RS (translated version)	Secure, insecure	Mother and father	PSS (translated version)	SA
Fattouh et al. 2022	Correlational study	Lebanon	1807	14–17 (15.42 ± 1.14)	Non‐clinical	RQ (translated version)	Secure, preoccupied, dismissing, fearful	General	C‐SSRS (translated version)	SI
Ding et al. 2022	Correlational study	China	4574	15–18 (16.28 ± 1.09)	Non‐clinical	IPPA‐12	Total score	Parent	Single‐item measure	SI
Guo et al. 2021	Correlational study	China	8680	12–19 (15.224 ± 2.559)	Non‐clinical	IPPA (translated version)	Total score	Parent and peer	PANSI (translated version)	SI
Hunt et al. 2021	Correlational study (data from RCT)	The United States	117	12–17 (14.89 ± 1.68)	Clinical	ECR‐RS	Anxious, avoidant	Mother and father	SIQ‐Jr	SI
Mirkovic et al. 2021	Between‐groups study	France, Belgium and Switzerland	75	15–19 (16.3 ± 1.4)	Clinical	RQ (translated version)	Secure, fearful, dismissing, preoccupied	General	Single‐item measure	SA
Herres et al. 2021	Correlational study (data from RCT)	The United States	129	12–18 (14.87 ± 1.68)	Clinical	ECR‐RS	Avoidant	Parent	SIQ‐Jr	SI
Hermosillo‐de‐la‐Torre et al. 2021	Between‐groups study	Mexico	8033	14–21 (16 ± 0.98)	Non‐clinical	CAMIR‐R (translated version)	Security, family preoccupation	Family	SBS	SA
Waraan et al. 2021	Correlational study (data from RCT)	Norway	50	13–17 (15 ± 1.3)	Clinical	ECR‐RS	Anxious and avoidant	Mother and father	SIQ‐Jr	SI
Potard et al. 2020	Correlational study	France	455	12–18 (15.78 ± 1.61)	Non‐clinical	IPPA (translated version)	Total, trust, communication, alienation	Mother and father	SIQ (translated version)	SI
Cantón‐Cortés et al. 2020	Correlational study	Spain	376	18–24 (19.55 ± 1.71)	Non‐clinical	ASM	Secure, anxious and avoidant	Parents	SSI	SI at present/crisis
Moyano et al. 2022	Correlational study	Ecuador	395	14–19 (15.86 ± 1.00)	Non‐clinical	IPPA‐15 (translated version)	Trust, communication and alienation	Mother, father and peer	Single‐item measure	SI
Chang et al. 2020	Correlational study (data from RCT)	The United States	128	12–18 (14.87 ± 1.68)	Clinical	ECR‐RS	Anxious and avoidant	Mother	SIQ‐Jr	SI
Handley et al. 2019	Correlational study (data from RCT)	The United States	164	(14 ± 0.85)	Non‐clinical	IPPA	Total score	Mother	Single‐item measure	SI
Cerutti et al. 2018	Correlational study	Italy	709	10–15 (12.6 ± 1.06)	Non‐clinical	IPPA (translated version)	Total score	Parent and peer	Single‐item measure	SI
Bar‐Zomer and Klomek 2018	Correlational study	Israel	279	10–17 (13.5 ± 1.9)	Non‐clinical	ASS	Secure	Mother and father	MFQ‐SI (4)	SI
Ibrahim et al. 2018	Correlational study (data from RCT)	The United States	115	13–18 (14.96 ± 1.68)	Clinical	ECR‐RS	Anxious and avoidant	Mother and father	SIQ‐Jr	SI
Sharif and Akhtar 2018	Correlational study	Pakistan	200	17–20	Non‐clinical	AAS	Secure, anxious, avoidant	General	BSI	SI
Nunes and Mota 2017	Correlational study	Portugal	604	15–18(15.99 ± 0.97)	Non‐clinical	FMAQ	Quality of emotional bond, inhibition of exploration and individuality and separation anxiety	Mother and father	SIQ (translated version)	SI
Zisk et al. 2017	Correlational study (data from RCT)	The United States	129	12–18 (14.96 ± 1.66)	Clinical	ECR‐RS	Anxious and avoidant	Mother	C‐SSRS (ISI)	SI
Sharaf et al. 2016	Correlational study	Egypt	150	13–21 (17.84 ± 1.97)	Clinical	PBI (translated version)	Care, overprotection	Mother and father	SIS (translated version)	SI
Lee 2016	Correlational study	South Korea	784	13–15 (14.38 ± 1.68)	Non‐clinical	IPPA, PBI (translated version)	Total score	Peer‐IPPA; mother and father‐PBI	Single‐item measure	SI
Li et al. 2016	Correlational study	China	1529	13–17 (14.74 ± 1.48)	Non‐clinical	IPPA‐13 (translated version)	Total score	Parent	Single‐item measure	SI, SA
Sheftall et al. 2014	Between‐groups study	The United States	80	13–18 (15.56 ± 1.35)	Clinical	ECR	Anxious and avoidant	General	SHF	SA
Saffer et al. 2015	Between‐groups study	The United States	598	(15.04 ± 1.39)	Non‐clinical + clinical	Modified version of PBI	Care, overprotection	Mother and father	Single‐item measure	SI, SA
Cruz et al. 2015	Between‐groups study	Portugal	42	13–21(16 ± 1.86)	Clinical	FMAQ	Quality of emotional bond, inhibition of exploration and individuality separation anxiety and dependence	Mother and father	Single‐item measure	SI, SA
Venta et al. 2014	Correlational study	The United States	133	12–17 (14.69 ± 1.478)	Clinical	KSS	Secure	Mother	Single‐item measure	SI
Venta and Sharp 2014	Between‐groups study	The United States	194	12–17 (16 ± 1.4)	Clinical	CAI	Secure, preoccupied, dismissing and disorganised	Caregivers	Single‐item measure	SI, SA
Boricevic Marsanic et al. 2014	Between‐groups study	Croatia	231	12–18 (15.26 ± 1.63)/(15.15 ± 1.46)	Clinical	PBI (translated version)	Care, control	Mother and father	Single‐item measure	SA
Sheftall et al. 2013	Between‐groups study	The United States	236	12–17 (14.48 ± 1.69/1.51)	Clinical	IPPA	Total score	Mother, father and peer	Interview	SA
Phuong et al. 2013	Correlational study	Vietnam	972	12–15	Non‐clinical	PBI	Care, overprotection	Mother and father	Single‐item measure	SI
Peltzer and Pengpid 2012	Correlational study	Thailand	2758	13–15	Non‐clinical	3‐item scale	Total score	Parent	Single‐item measure	SI
Maimon et al. 2010	Longitudinal study (3 years)	The United States	990	11–16 (13.5 ± 1.52)	Non‐clinical	6‐item scale	Total score	Family	Single‐item measure	SA
Maimon and Kuhl 2008	Longitudinal study (2 years)	The United States	6369	12–21 (16.3 ± 1.62)	Non‐clinical	4‐item scale	Total score	Mother and father	Single‐item measure	SA
Peter et al. 2008	Correlational study (data from longitudinal study)	Canada	1032	12–15 (13.6 ± 1.1)	Non‐clinical	20‐item (parent), 4‐item (peer) scale	Positive stimuli, negative stimuli, parental involvement and total score (peer)	Parent and peer	Single‐item measure	SI
Kidd and Shahar 2008	Correlational study	The United States and Canada	208	14–24 (20.25 ± 2.39)	Non‐clinical	RQ	Secure, preoccupied, dismissing and fearful	General	4‐item scale	SI
Nrugham et al. 2008	Longitudinal study (6 years)	Norway	265	13–14 (13.7 ± 0.5)	Non‐clinical	IPPA, IPPA‐6 (peer) (translated version)	Trust, communication and alienation (mother and father); peer total score	Mother, father and friend	Single‐item measure	SA
Silviken and Kvernmo 2007	Between‐groups study (data from longitudinal study)	Norway	2691	16–18 (16.9 ± 0.8)	Non‐clinical	PBI (translated version)	Care, overprotection	Mother and father	Single‐item measure	SA
Lai and McBride‐Chang 2001	Between‐groups study	China	120	15–19 (16.1 ± 1.6)	Non‐clinical	PBI (translated version)	Care, overprotection	Mother and father	SSI (translated version)	SI
DiFilippo and Overholser 2000	Correlational study	The United States	59	13–17 (15.6 ± 1.2)	Clinical	IPPA	Total score	Mother, father and peer	BSI	SI
Fergusson et al. 2000	Longitudinal study (6 years)	New Zealand	1265	15–21	Non‐clinical	IPPA	Total score	Parent	Single‐item measure	SI, SA
Lessard and Moretti 1998	Correlational study	Canada	116	10–17 (13.5 ± 1.4)	Clinical	Semi‐structured interview	Secure, fearful, preoccupied and dismissing	Caregivers	Single‐item measure	SI
Beautrais et al. 1996	Between‐groups study	The United Kingdom	282	13–24 (21.4 ± 1.6)	Non‐clinical + clinical	PBI	Care	Father	Medical record data	SA
Adam et al. 1994	Between‐groups study	Canada	187	12–19 (14.9 ± 1.6)	Clinical	PBI	Care, overprotection	Mother and father	Interview	SI, SA
Martin and Waite 1994	Between‐groups study	Australia	681	14–18 (15 ± 0.6)	Non‐clinical	PBI	Care, protection	Mother and father	Single‐item measure	SI
Strang and Orlofsky 1990	Between‐groups study	The United States	191	College student under 21 (median is 19.5)	Non‐clinical	IPPA	Total score	Parents and peers	SSI	SI

*Note:* *Suicidal thoughts and behaviours measures: C‐SSRS: Columbia‐Suicide Severity Rating Scale, SIQ: Suicide Ideation Questionnaire, SIQ‐Jr: Suicidal Ideation Questionnaire‐Junior, MFQ‐SI (4/5): Mood and Feelings Questionnaire (four or five items related to suicidal ideation).

Abbreviations: *SI, suicidal ideation; AAS, Adult Attachment Scale; ASM, Attachment Style Measure; ASS, Attachment Security Style Scale; Attachment measures: RQ, Relationship Questionnaire; BSI, Beck Scale for Suicide Ideation; CAI, Child Attachment Interview; CAMIR‐R, Questionnaire of Attachment Evaluation; C‐SSRS (ISI), Intensity of Ideation subscale of the Columbia‐Suicide Severity Rating Scale; ECR, Experiences in Close Relationships; ECR‐RS, Experiences in Close Relationships‐Relationships Structure questionnaire; FMAQ, Father/Mother Attachment Questionnaire; IPPA, Inventory of Parent and Peer Attachment; KSS, the Security Scale; PANSI, Positive and Negative Suicide Ideation inventory; PBI, Parental Bonding Instrument; PSS, Paykel Suicide Scale; SA, suicide attempt; SBS, Suicidal Behaviours Schedule (Cédula de Conductas Suicidas); SHF: Columbia University Suicide History Form; Short version of IPPA, IPPA‐6 (6 items), IPPA‐12, IPPA‐13, IPPA‐15, IPPA‐20, IPPA‐9; SIS, Suicide Intent Scale; SSI, Scale for Suicide Ideation; SSOSI, Self‐rating scale of suicidal ideation.

### 4.3 Quality Assessment

Following the quality assessment tool's guidance (National Heart, Lung, and Blood Institute (NHLBI), and National Institutes of Health (NIH), n.d.), 34 studies were rated as *Good* quality (low risk of bias), 17 as *Fair* quality (some risk of bias) and 3 as *Poor* (high level of bias). Regarding the 17 *Fair* studies, one was rated as *Fair* due to a low follow‐up rate (< 60%). Three studies received a *Fair* rating because they did not account for potential confounders. The other 13 studies received a *Fair* rating because they did not utilise validated attachment measures, including unvalidated translated versions (*n* = 9) and the use of 4–6 idiosyncratic items (*n* = 4), potentially overlooking key aspects of attachment. Of the three ‘poor’ studies, two (Lai and McBride‐Chang 2001; Lessard and Moretti 1998) failed control for confounding factors and used unvalidated attachment measures. The other study (Sharif and Akhtar 2018) lacked detail in recruitment and selection procedures and overlooked potential confounders. Full quality assessment results and detailed justifications are provided in Appendix S4.

### 4.4 Findings From Meta‐Analysis

To examine the relationship between attachment concepts and SI or SA, seven separate meta‐analyses were conducted. Subgroup analyses were restricted to subgroups with data from five or more studies to ensure sufficient power (Jackson and Turner 2017). These subgroup meta‐analyses examined the associations between: (a) attachment security and SI; (b) attachment avoidance and SI; (c) attachment anxiety and SI; (d) attachment quality (IPPA total score) and SI; (e) parental care and SI; (f) parental overprotection and SI; and (g) parental care and SA.

#### 4.4.1 Overall Relationship Between Attachment Concepts and SI or SA

Table 3 summarises the results for each subgroup meta‐analysis, and the associated forest plots display the pooled effect size (see Appendix S5). SI was shown to be significantly associated with higher attachment anxiety (*r* = 0.198, 95% CI: (0.139, 0.255)), higher parental overprotection (*r* = 0.108, 95% CI: (0.050, 0.164)), lower attachment security (*r* = −0.161, 95% CI: (−0.270, −0.049)), poorer attachment quality (*r* = −0.342, 95% CI: (−0.440, −0.236)) and lower parental care (*r* = −0.219, 95% CI: (−0.303, −0.132)), with attachment quality yielding the largest effect size. No significant association was found between SI and attachment avoidance (*r* = 0.061, 95% CI: (−0.057, 0.177)). Consistent with findings for SI, SA was significantly related to lower parental care (*r* = −0.279, 95% CI: (−0.393, −0.157)), with a medium effect size.

**TABLE 3:** Subgroup meta‐analysis.

Subgroup meta‐analysis	Total pooled sample size	Heterogeneity Q statistic			Publication bias (Egger's test)		Random effects meta‐analysis				
		*Q*‐value (df)	*p*	I square	*t*‐value (df)	*p*	*r*	95% CI		*Z*‐value	*p*
Attachment security–suicidal ideation	1033	16.867 (5)	0.005	70.356	1.88 (4)	0.301	−0.161	−0.27	−0.049	−2.799	0.005
Attachment avoidance–suicidal ideation	1079	21.855 (6)	0.001	72.547	0.108 (5)	0.919	0.061	−0.057	0.177	1.016	0.31
Attachment anxiety‐suicidal ideation	1078	6.028 (6)	0.42	0.471	0.464 (5)	0.662	0.198	0.139	0.255	6.494	< 0.001
Attachment quality‐suicidal ideation	17,565	392.653 (9)	< 0.001	97.708	0.690 (8)	0.510	−0.342	−0.44	−0.236	−6.027	< 0.001
Parental care‐suicidal ideation	18,976	229.756 (8)	< 0.001	96.518	0.914 (7)	0.391	−0.219	−0.303	−0.132	−4.829	< 0.001
Parental overprotection‐suicidal ideation	12,421	39.591 (7)	< 0.001	82.319	3.672 (6)	0.010	0.108	0.05	0.164	3.678	< 0.001
Parental care‐suicide attempts	11,168	102.214 (6)	< 0.001	94.130	2.753 (5)	0.040	−0.279	−0.393	−0.157	−4.385	< 0.001

#### 4.4.2 Heterogeneity

Heterogeneity analyses showed significant variability in most subgroups, including attachment security‐SI, attachment avoidance‐SI, attachment quality‐SI, parental care‐SI, parental overprotection‐SI and parental care‐SA (see Table 3).

#### 4.4.3 Publication Bias and Sensitivity Analysis

Egger's test was only significant for the parental overprotection‐SI and parental care‐SA groups, indicating possible publication or selection bias in these groups (See Table 3). Given the limited number of studies, Egger's test results may be unreliable (Egger et al. 1997). Therefore, funnel plots were also visually inspected. Asymmetry was observed in the parental care‐SI, parental overprotection‐SI and parental care‐SA groups, suggesting possible publication or selection bias (see Appendix S6).

The Trim‐and‐Fill method was used to detect and adjust for potential publication bias by estimating effects with imputed missing studies (Higgins et al. 2023). Adjusted estimates differed from observed ones in the parental overprotection‐SI group. After adjustment, the imputed estimates significantly decreased for parental overprotection‐SI (*r* = 0.047, 95% CI (−0.011, 0.105)) (see Appendix S7). These findings suggested that publication bias may have particularly affected the results in the parental overprotection‐SI group, and conclusions drawn from this subgroup should be interpreted with caution.

One‐study‐removed analyses showed no undue influence for most subgroups. Appendix S8 presents the pooled effects for each subgroup meta‐analysis, recalculated after excluding each study in turn. However, in the attachment avoidance group, removing the study by Lessard and Moretti (1998) resulted in a significant association (*r* = 0.104, 95% CI (0.020, 0.187), *p* = 0.015), suggesting a small positive association between avoidant attachment and SI. This study, rated as ‘poor’ due to using an unvalidated semi‐structured attachment interview, may have impacted the validity of the study findings. It is worth noting that although removing Lessard and Moretti (1998) altered statistical significance, the findings in the other studies remained inconsistent. Among the other studies conducted across both clinical and non‐clinical samples, two reported significant associations, whereas three reported nonsignificant effects. The relationship between avoidant attachment and suicidal ideation should therefore be interpreted with caution, and further research is needed to clarify this association. Eleven studies included in the meta‐analysis were rated as ‘fair’ or ‘poor’. Removing these studies had minimal impact on pooled estimates, indicating study quality did not substantially affect overall conclusions.

#### 4.4.4 Summary

The meta‐analysis found that SI was significantly associated with lower secure attachment, higher anxious attachment, poorer attachment quality, lower parental care and higher parental overprotection. SA was also significantly related to lower parental care. In contrast, avoidant attachment was not significantly associated with SI.

### 4.5 Narrative Synthesis

This section presents a narrative synthesis of results from studies included in the systematic review but excluded from the meta‐analyses due to insufficient data to allow conversion to effect size r or insufficient number of studies within specific subgroups (minimum of 5 studies). Relationships between attachment, SI and SA are summarised according to the previously defined four domains.

#### 4.5.1 Secure, Avoidant, Anxious, Disorganised Attachment

##### 4.5.1.1 Secure Attachment

Six studies measuring secure attachment were not included in the meta‐analysis. Two studies (Fattouh et al. 2022; Bar‐Zomer and Klomek 2018), both conducted with non‐clinical samples, found a significant negative association between attachment security and SI. The other four studies (Hermosillo‐De‐La‐Torre et al. 2021; Lara Leben Novak et al. 2023; Mirkovic et al. 2021; Myerson et al. 2023) investigated the association between attachment security and SA. Three studies, conducted in clinical and non‐clinical samples, reported significant negative associations, with small to medium effect sizes (*r* = −0.094 to −0.325). In contrast, Novak et al. (Lara Leben Novak et al. 2023) found paternal security to be significantly related to SA (*r* = −0.164), while maternal security was not (*r* = −0.007). Overall, findings support an association between secure attachment and SA, consistent with meta‐analysis results for SI.

##### 4.5.1.2 Avoidant Attachment

This section includes studies reporting both ‘avoidant attachment’ and ‘dismissing attachment’, as these two labels have been used interchangeably in the literature (Ravitz et al. 2010).

Four studies examined attachment avoidance in relation to SI (*n* = 1) or SA (*n* = 3).

Fattouh et al. (2022) reported a significant positive association between SI and avoidant attachment in non‐clinical samples, with a moderately large effect (B = 0.528, 95% CI: (0.285, 0.772)).

For SA, findings were mixed, echoing the meta‐analysis results. Specifically, Mirkovic et al. (2021) reported a medium negative association (*r* = −0.237, 95% CI: (−0.431, −0.022)), Sheftall et al. (2014) suggested a medium positive association (*r* = 0.351, 95% CI: (0.158, 0.518)), Myerson et al. (2023) found no association (*r* = 0.036, 95% CI: (−0.039, 0.110)). All three studies were conducted in clinical samples.

Overall, the study on SI found a positive association between avoidant attachment and SI, contrasting with the meta‐analysis. Findings on SA remain inconclusive, and the relationship between avoidant attachment and SA was excluded from the meta‐analysis due to insufficient studies (*n* < 5).

##### 4.5.1.3 Anxious Attachment

This section includes studies investigating ‘anxious attachment’ and ‘preoccupied attachment’, given the interchanging use of these two labels within the literature (Ravitz et al. 2010). One study explored the relationship between anxious attachment and SI (Fattouh et al. 2022), and four studies examined the relationship between anxious attachment and SA (Sheftall et al. 2014; Myerson et al. 2023; Hermosillo‐de‐la‐Torre et al. 2021; Mirkovic et al. 2021).

Fattouh et al. (2022) found no significant association between anxious attachment and SI, differing from the meta‐analysis.

In terms of the relationship between anxious attachment and SA, four studies (Sheftall et al. 2014; Myerson et al. 2023; Hermosillo‐de‐la‐Torre et al. 2021; Mirkovic et al. 2021) reported small, nonsignificant effects (*r* ranging from −0.008 to 0.155) across clinical and non‐clinical samples.

In summary, the narrative synthesis suggests that anxious attachment is not associated with SI. Any associations between SA and attachment are likely to be weaker and not maintained when controlling for confounds.

##### 4.5.1.4 Disorganised Attachment

The concept of fearful attachment shares similar psychological characteristics with the concept of disorganised attachment (Ravitz et al. 2010), so are discussed together. Three studies (Lessard and Moretti 1998; Fattouh et al. 2022; Kidd and Shahar 2008) examined the relationship between disorganised attachment and SI. Two of these studies (Lessard and Moretti 1998; Kidd and Shahar 2008) indicated a small to medium significant positive association (*r* = 0.23 and 0.32), drawing on clinical and non‐clinical samples. In contrast, Fattouh et al. (2022) found no significant difference in SI between individuals with low versus high disorganised attachment in non‐clinical samples.

Two studies in clinical samples examined the relationship between disorganised attachment and SA (Myerson et al. 2023; Mirkovic et al. 2021), both showing small, nonsignificant positive effects (*r* = 0.019 and 0.070).

Overall, disorganised attachment was associated with SI but not with SA.

##### 4.5.1.5 Comparing Attachment Styles (Secure, Anxious, Avoidant and Disorganised)

Venta and Sharp (2014) were the only study that used a categorical classification, assigning each participant to a single attachment category based on the highest of the four dimensional ratings. This study focused on identifying which specific attachment style is most closely associated with SI or SA among clinical participants. They found no specific attachment style was significantly more strongly linked to SI or SA when compared with other attachment styles (*p* > 0.05), suggesting that individual attachment styles cannot reliably identify those at higher risk.

#### 4.5.2 Trust, Communication, Alienation, Attachment Quality

The IPPA is a widely used measure of self‐reported attachment with parents or peers, assessed across trust, communication and alienation, with overall quality based on these dimensions (Armsden and Greenberg 1987).

Six studies reporting IPPA‐based attachment quality were excluded from the meta‐analysis: five assessed parental attachment and five peer attachment. Four studies conducted in clinical and non‐clinical samples examining parental attachment found a significant association with SI (Fergusson et al. 2000; Bakken et al. 2024; DiFilippo and Overholser 2000) or SA (Fergusson et al. 2000; Sheftall et al. 2013), consistent with meta‐analytic findings. Notably, Fergusson et al. (2000) and Bakken et al. (2024) employed longitudinal data in non‐clinical samples, demonstrating that early attachment quality predicted later SI (Fergusson et al. 2000; Bakken et al. 2024) and SA (Fergusson et al. 2000) over follow‐up periods ranging from 6 to 13 years, suggesting temporal relationships. Bakken et al. (2025) analysed earlier waves (baseline and Time 1) of the same longitudinal dataset as Bakken et al. (2024) (Time 1 and Time 3), and found no significant associations between parental attachment quality and SI or SA.

Among five studies on peer attachment, both clinical and non‐clinical samples were included. Four studies suggested peer attachment quality was significantly related to SI (Bakken et al. 2024; DiFilippo and Overholser 2000), but not to SA (Sheftall et al. 2013; Nrugham et al. 2008), suggesting peer attachment may be more relevant to ideation than attempts. However, Bakken et al. (2025) found no significant associations between peer attachment quality and either SI or SA.

Overall, IPPA‐based parental and peer attachment quality was linked to SI, consistent with meta‐analysis. SA was related to parental attachment quality but not to peer attachment. Regarding IPPA subscales, studies (Strang and Orlofsky 1990; Potard et al. 2020; Moyano et al. 2022; Guo et al. 2023; Shin and Bae 2024) based on non‐clinical samples showed trust and communication negatively associated with SI, while alienation was positively associated. Parental attachment subscales, especially trust and communication, showed stronger links to SI than peer subscales. However, Nrugham et al. (2008), also employing a non‐clinical sample, found no substantial associations between any parental attachment IPPA subscale and SA.

#### 4.5.3 Care and Overprotection

The PBI is a frequently used retrospective self‐report scale that measures individuals' perceptions of bonding with their parents within the first 16 years of life (Parker et al. 1979). It assesses attachment along two dimensions derived from attachment theory: care and overprotection (Parker et al. 1979).

Across four studies with varied sample characteristics (clinical and community samples; diverse ethnic groups), parental overprotection was consistently positively associated with SA. Lee (2016) reported total PBI scores found a negative correlation with SI, indicating poor parenting experiences were linked with higher SI. Expanding on meta‐analysis, Cohen and Stutts (2023) and Saffer et al. (2015) found adolescents with SI alone had higher care scores than those who attempted suicide. Adam et al. (1994) compared total PBI scores across NSSI, SI and SA groups, finding the NSSI group scored highest.

In summary, the narrative synthesis suggests a positive association between parental overprotection and SA. Parental care may help distinguish SA from SI, and total PBI scores may differentiate those with STB from those without.

#### 4.5.4 Other Attachment Concepts Assessed Using Other Scales

Six studies, comprising both cross‐sectional and longitudinal designs, used other measures to assess attachment.

Two cross‐sectional studies (Cruz et al. 2015; Nunes and Mota 2017) assessed attachment with the Father/Mother Attachment Questionnaire (FMAQ), which measures quality of emotional bond (QEB), separation anxiety and dependence (SAD) and inhibition of exploration and individuality (IEI) (Matos et al. 2001). Findings were inconsistent. Nunes and Mota (2017), based on non‐clinical samples, found QEB negatively associated with SI, while SAD and IEI were positively associated. Cruz et al. (2015) found no significant association between FMAQ‐based attachment concepts and SI or SA.

Four studies assessed attachment with 4–6 idiosyncratic items. Two (Peltzer and Pengpid 2012; Peter et al. 2008) were cross sectional and two (Maimon and Kuhl 2008; Maimon et al. 2010) were longitudinal, with all studies conducted in non‐clinical settings. Both cross‐sectional studies found that adolescent SI was associated with negative parental attachment. Both longitudinal studies found that poor family attachment predicted later SA. Specifically, a one‐unit increase in positive attachment to parents or family was associated with a 21% (Maimon and Kuhl 2008) to 35% (Maimon et al. 2010) reduction in the likelihood of future SA.

### 4.6 Factors That Influence the Relationships

#### 4.6.1 Mediators

##### 4.6.1.1 SI

Eight studies investigated mediators of the relationship between attachment and SI. Five studies in non‐clinical samples examined intrapersonal mediators, referring to variables internal to the individual, and three studies (one in non‐clinical and two in clinical samples) examined interpersonal mediators, referring to factors involving interpersonal interactions.

Intrapersonal mediators included trait anhedonia (Guo et al. 2021), difficulty in identifying and describing feelings (Cerutti et al. 2018), separation anxiety (Potard et al. 2020), depression and impulsivity (Moyano et al. 2022) and resilience (Sharif and Akhtar 2018). Of these mediators, only resilience did not show a mediating effect.

Interpersonal mediators included perceived burdensomeness (Hunt et al. 2021; Venta et al. 2014), thwarted belongingness (Venta et al. 2014) and peer attachment quality (Guo et al. 2021) (viewed as peer interactions shaped by parental attachment quality, as early perception of caregiver relationships shape how individuals perceive and interact within peer relationships). Thwarted belongingness (Venta et al. 2014) (B = −0.386, *p* < 0.001) and peer attachment (Guo et al. 2021) (B = −0.010, *p* < 0.01) showed mediating effects.

Findings on perceived burdensomeness were inconsistent (Hunt et al. 2021; Venta et al. 2014). Hunt et al. (2021) found a mediating effect only between attachment anxiety and SI, not attachment avoidance. Venta et al. (2014) found no significant mediating effect of perceived burdensomeness.

##### 4.6.1.2 SA

Two studies among clinical participants examined mediators of the relationship between attachment and SA: emotion dysregulation (Mirkovic et al. 2021) and the acquired capability for suicide (ACS) (Lara Leben Novak et al. 2023). The ACS showed a mediating effect, while emotion dysregulation did not.

Mirkovic et al. (2021) found no significant relationship between insecure attachment and emotion dysregulation (B = 0.016, *p* = 0.219), resulting in no mediating effect.

Lara Leben Novak et al. (2023) found that ACS mediated the relationship between attachment avoidance and SA (B = 0.21 for paternal, B = 0.23 for maternal, *p* < 0.01). The ACS also mediated the relationship between paternal attachment anxiety and SA (B = −0.17, *p* < 0.01), but no mediation was found for maternal attachment anxiety.

However, all studies examining mediators used cross‐sectional designs. Longitudinal or microlongitudinal research is needed to clarify directionality and potential causality. Heterogeneity in measured concepts also limited integration and interpretation of findings.

#### 4.6.2 Moderators

##### 4.6.2.1 SI

Five studies investigated moderators of the relationship between insecure attachment and SI. Factors examined included gender (DiFilippo and Overholser 2000; Potard et al. 2020; Moyano et al. 2022), social involvement (Kidd and Shahar 2008), self‐esteem (Kidd and Shahar 2008) and environmental sensitivity (Dong et al. 2024). Among these factors, only environmental sensitivity (Dong et al. 2024) showed a moderating effect. Dong et al. (2024) found that environmental sensitivity, defined as individuals' capacity to perceive and respond to environmental influences, significantly moderated the relationship between parental care and SI. Highly sensitive adolescents benefited more from increased parental care, which helped protect against SI. Conversely, reduced parental care may heighten their risk.

##### 4.6.2.2 SA

One study examined whether neighbourhood collective efficacy, defined as informal control and regulation to achieve common well‐being, moderated the relationship between attachment and SA. Maimon et al. (2010) found that collective efficacy significantly and negatively moderated the relationship between family attachment and SA (B = −0.186, *p* < 0.05), with the effect significant only when collective efficacy was high.

## 5 Discussion

This systematic review and meta‐analysis aimed to (a) evaluate evidence quality; (b) evaluate the strength of the relationship between attachment concepts and SI or SA in adolescents through subgroup meta‐analyses; (c) provide narrative synthesis of available evidence; (d) examine mediators and moderators of these relationships; and (e) offer recommendations for future research and clinical practice. The review identified 54 studies, including 27 with extractable effect sizes.

### 5.1 Study Quality

Overall, the quality of studies was good. A smaller number were rated as ‘fair’ or ‘poor’, mainly due to the use of unvalidated attachment measures or unvalidated translated versions. The quality assessment followed established criteria requiring evidence of validation. Therefore, studies without such evidence were rated as ‘fair’ or ‘poor’ to reflect methodological uncertainty rather than conceptual inadequacy. Although unvalidated attachment measures or unvalidated translated versions capture relevant attachment constructs, the lack of validation warranted a cautious approach in quality rating. However, because unvalidated translated measures are used predominantly in non‐English samples, this criterion may place such studies at a systematic disadvantage in quality assessments. Therefore, lower ratings for unvalidated translated measures can be understood as reflecting methodological uncertainty arising from limited instrument availability rather than evidence of methodological limitations. This interpretation helps avoid overstating the disadvantage faced by these studies.

In the meta‐analyses, study quality did not influence the overall conclusions. In the narrative synthesis, discrepancies may stem from the use of unvalidated measures, particularly translated versions that lacked formal validation. Notably, studies rated ‘fair’ (Fattouh et al. 2022; Lara Leben Novak et al. 2023) that used such measures reported nonsignificant results, contrasting with the significant findings in ‘good’ studies. These findings highlight the need for properly validated attachment measures, especially in translation, to address cultural and linguistic differences. However, nonsignificant findings in studies conducted with non‐English samples may also reflect genuine cultural differences in caregiving, attachment, and STB, rather than translation issues alone (Chu et al. 2010; van IJzendoorn and Kroonenberg 1988). Therefore, culturally sensitive and psychometrically validated attachment and STB measures are needed to determine whether such nonsignificant findings reflect true cultural variation or limitations in measurement validity.

### 5.2 Attachment Concepts: SI and SA

The findings indicated that positive attachment concepts, such as secure attachment, positive attachment quality and parental care, were negatively associated with SI, while negative attachment concepts, including anxious attachment, disorganised attachment and parental overprotection, showed positive associations. In contrast, avoidant attachment showed no significant relationship with SI. Similar patterns were observed for SA, although anxious and disorganised attachment showed weaker associations than with SI. Longitudinal studies (Fergusson et al. 2000; Bakken et al. 2024; Maimon and Kuhl 2008; Maimon et al. 2010) suggested that early negative attachment experiences can increase the likelihood of later SI and SA. These associations did not seem to be influenced by sample type (clinical vs. community). However, other aspects of study design may influence the association between attachment concepts and SI or SA. A small number of studies reported contrasting findings on the relationship between attachment concepts and SI or SA. These mixed findings may reflect heterogeneity in study design, including sample size (Cruz et al. 2015), gender composition (Nrugham et al. 2008), family structure (Lara Leben Novak et al. 2023), cultural context (Fattouh et al. 2022) and measures of attachment constructs (Lessard and Moretti 1998; Fattouh et al. 2022; Cohen and Stutts 2023; Saffer et al. 2015; Boricevic Marsanic et al. 2014; Silviken and Kvernmo 2007). Given the limited number of studies within each subgroup, these design factors were not formally tested as moderators of the observed associations. Future work could benefit from moderation analyses to evaluate whether such design features help to explain variation in the observed associations.

Adolescents are beginning to navigate relationships beyond the family and are still developing interpersonal skills, which may increase exposure to interpersonal difficulties and related distress (Steinberg and Morris 2001). Therefore, adolescents may be more vulnerable to STB than adults. Adolescents with insecure attachment may struggle to regulate emotions when interpersonal difficulties arise (Bowlby 1969; Mikulincer and Shaver 2019). They may also process social information in less adaptive ways that can intensify relationship problems (Bowlby 1969; Crick and Dodge 1994). Therefore, insecure attachment may increase adolescents' risk of STB. These findings may inform clinical practice by highlighting the importance of assessing potential suicidality in adolescents with insecure attachments. Overall, findings support Adam's model of suicide, which proposes that early adverse attachment experiences increase vulnerability to later STB (Adam 1994). However, this model does not distinguish pathways to SI versus SA, limiting its specificity.

The lack of significant relationships between attachment avoidance and SI or SA is consistent with prior reviews in adolescents and adults (Miniati et al. 2017; Zortea et al. 2021; Woo et al. 2022). This intriguing result may reflect reluctance among avoidantly attached adolescents to disclose SI, due to distrust of others and the use of deactivating strategies that suppress negative affect (Waraan et al. 2021; Ibrahim et al. 2018). As a result, such adolescents may conceal SI or struggle to bring it into conscious awareness during self‐report assessments (Mikulincer and Nachshon 1991).

This review also found no evidence for a relationship between anxious attachment and SA, consistent with a previous adolescent review (Woo et al. 2022), but contrasting with adult‐focused reviews (Miniati et al. 2017; Zortea et al. 2021). These differences may reflect age‐related variations in coping. Adolescents with anxious attachment often seek support during distress, which may protect against progression from ideation to attempts (Mikulincer and Shaver 2019; Mikulincer and Shaver 2010). In contrast, adults may be more independent and less likely to seek help (Knoll et al. 2017; Loeb et al. 2021), increasing risk. In addition, continued involvement in family and school environments during adolescence can also increase access to potential sources of support and opportunities for detection and intervention, compared with adulthood. Furthermore, both this review and the adolescent‐focused review included only four studies on this association, highlighting the need for further research.

### 5.3 Mediators and Moderators

#### 5.3.1 Mediators

This review identified emotional and interpersonal factors as potential mediators between attachment and SI. Adam's model of suicide has been extensively examined in adults (Green et al. 2020) but has remained unexplored in adolescents. According to this model, early adverse attachment experiences negatively impact self‐worth, emotional regulation and interpersonal functioning, thereby increasing vulnerability to SI and SA (Adam 1994). The findings support this pathway by highlighting emotional and interpersonal processes as key mechanisms linking attachment to SI. However, one critical mediator, self‐worth, was not investigated in the reviewed studies. Adolescence is a critical stage for identity formation and self‐evaluation (Crone and Fuligni 2020). During this period, the development and maintenance of self‐worth are closely linked to adolescents' psychological well‐being and to STB (Bırni and Eryılmaz 2024). Therefore, further research is needed to explore the mediating role of self‐worth, as supported by findings in the adult literature (Imran and Jackson 2022; Zortea et al. 2019).

The results also align with Joiner's Interpersonal‐Psychological Theory (IPT) (Joiner 2005). This model posits that thwarted belongingness and perceived burdensomeness contribute to SI, while ACS increases risk of attempts (Joiner 2005). Consistent with IPT, this review found that thwarted belongingness and perceived burdensomeness mediate the association between insecure attachment and SI, and ACS mediates between insecure attachment and SA. These findings suggested the IPT is applicable to adolescents. As young people begin to form close peer relationships during adolescence, adolescents' IWMs guide expectations and behaviour in these relationships (Bowlby 1969). Adolescents with insecure attachment hold negative perceptions of self and others, making it hard to build and maintain peer connections (Bowlby 1969). Limited connection with peers can reduce feelings of belongingness and increase social disconnection, which may be experienced as thwarted belongingness (Kirshenbaum et al. 2024). They may also interpret difficulties in forming peer relationships as evidence that they are a burden to others, contributing to perceived burdensomeness (Kirshenbaum et al. 2024). Thwarted belongingness combined with perceived burdensomeness increases the risk of SI (Van Orden et al. 2010).

However, some exceptions emerged. Certain studies found no significant link between avoidant attachment and perceived burdensomeness (Hunt et al. 2021), and no association between maternal attachment anxiety and ACS (Lara Leben Novak et al. 2023). These discrepancies may partly reflect different attachment strategies. Avoidant attachment, with deactivating strategies and high self‐worth, may reduce feeling like a burden (Mikulincer and Shaver 2012). Anxious attachment, with hyperactivating strategies and low self‐worth, may lead to support‐seeking, which could lower the risk of attempts and shows a weaker link to ACS (Mikulincer and Shaver 2019). These findings suggested that distinct attachment patterns may associate with STB through different pathways, highlighting the need for tailored intervention strategies.

Furthermore, all studies examining mediators used cross‐sectional designs. Longitudinal or microlongitudinal research is needed to clarify directionality and potential causality.

#### 5.3.2 Moderators

This review found that both environmental sensitivity and neighbourhood collective efficacy moderated the relationship between attachment and SI or SA. According to the Integrated Motivational‐Volitional (IWV) Model, individuals with high environmental sensitivity show heightened responsiveness to negative environmental signals (O'Connor and Kirtley 2018; Kirtley et al. 2015). As a result, they may be more likely to experience feelings of defeat when facing adverse attachment experiences. Such feelings of defeat are a key driver in the pathway towards STB (O'Connor and Kirtley 2018; Kirtley et al. 2015). On the other hand, heightened sensitivity may also amplify the benefits of positive attachment experiences, thereby reducing the risk of STB (Colich et al. 2021). Self‐identity formation and belonging‐seeking are central developmental tasks during adolescence (Steinberg and Morris 2001). Adolescents therefore pay close attention to feedback from social environments (family, school and peers) and can be highly responsive to social signals. When this adolescence‐specific sensitivity to social feedback combines with high environmental sensitivity, negative attachment experiences may heighten their suicide risk, whereas positive ones may mitigate it (Colich et al. 2021). The IMV Model also highlights social support as a protective factor that buffers against the development of STB (O'Connor and Kirtley 2018). Social support builds feelings of being supported by others and helps individuals move out of trapped negative states. This process contributes to a reduction in STB (O'Connor and Kirtley 2018). The moderating role of neighbourhood collective efficacy, which is related to social support (Sampson et al. 1997; Vassilev et al. 2019), aligns with this model. Compared with adults, adolescents spend more time within their families and in school settings. These findings therefore highlight the importance of promoting positive family and school environments and enhancing social support within the broader community. The lack of a significant moderating effect of gender aligned with findings from a review in adults that examined the relationship between attachment and STB (Zortea et al. 2021). Taken together, findings from the adult review and the present review may suggest that the relationship between insecure attachment and SI does not differ substantially across genders. However, given the limited number of studies, further investigation is needed.

**FIGURE 3:**
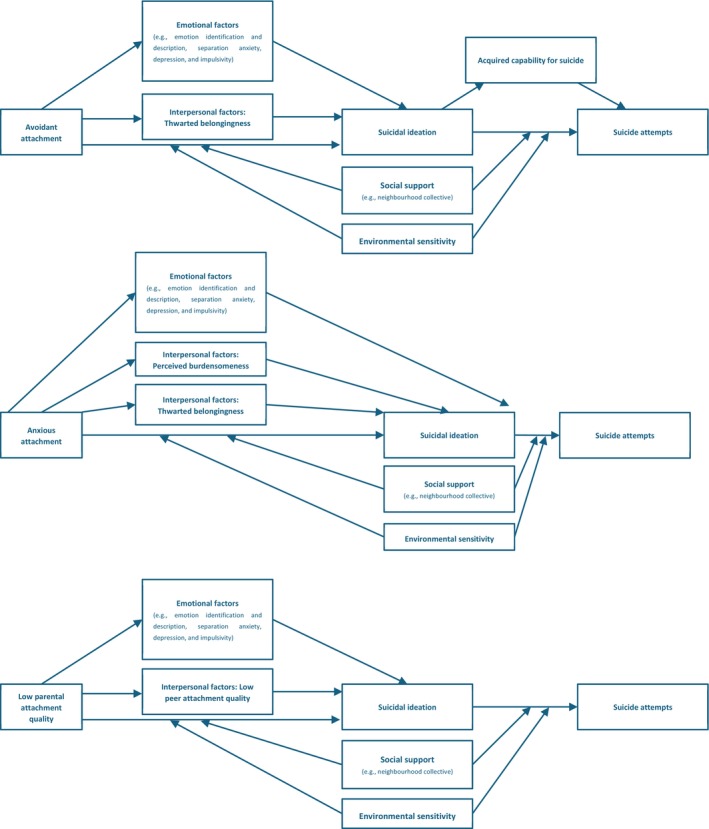
An integrated conceptual framework linking attachment concepts to suicidal thoughts and behaviours in adolescents. Note: Arrows represent hypothesised associations only and are not indicative of causal direction, as the included studies are cross sectional.

Based on the mediator and moderator findings in the included studies, Figure 3 summarises an integrated conceptual framework linking attachment concepts to STB in adolescents. Attachment‐related concepts measured by the IPPA and PBI (i.e., perceived relationship quality) are theoretically shaped by adolescents' IWMs that underlie attachment styles. However, none of the included studies assessed attachment styles alongside IPPA/PBI constructs and explored whether these constructs could help explain the association between attachment styles and STB. Therefore, Figure 3 does not illustrate how attachment styles may relate to STB through these attachment‐related concepts. Future research would benefit from integrating attachment styles with attachment‐related concepts to test whether attachment‐related concepts mediate the association between attachment styles and STB.

### 5.4 Strengths and Limitation

It is important to consider the strengths and limitations of this review. A key strength is its use of meta‐analysis to estimate the effect sizes of specific attachment concepts on SI in adolescents. The inclusion of a large number of studies and the combination of narrative synthesis and meta‐analysis provided a comprehensive overview of the evidence on attachment concepts and SI or SA.

However, there are also some limitations to this review. The author team is proficient in English and Chinese, and therefore, the review focused on studies published in these two languages (although no studies in Chinese were included due to ineligibility). Studies (*n* = 18) published in languages other than English or Chinese (authors' languages) were excluded. These studies were excluded due to limited information in the non‐English/Chinese titles and abstracts, and it was unclear whether they met the inclusion criteria. This exclusion may have led to the absence of relevant research. Using professional translation services for multiple languages would have been challenging, and translation platforms could introduce inaccuracies, compromising the accuracy of the data. In addition, over 80% of the included studies were conducted in Western countries, reflecting a historical Western bias in this area of research. Cultural norms and stigma related to mental health and STB may influence individuals' willingness to disclose STB (Chu et al. 2010). In addition, cross‐cultural differences may shape the development and expression of attachment styles, with avoidant attachment more commonly observed in individualistic societies and anxious attachment in collectivistic ones (van IJzendoorn and Kroonenberg 1988). As a result, the findings may not fully capture the experiences of individuals from culturally diverse backgrounds. Conducting additional research in Eastern countries, and with peoples from the global majority, could enhance the generalisability of the findings. It would also offer a more comprehensive understanding of how attachment relates to STB across diverse cultural contexts, helping to explore potential cross‐cultural differences.

To ensure methodological rigour and consistency, only peer‐reviewed studies were included. Unpublished studies were excluded as they had not undergone a formal peer‐review process, and their inclusion may have introduced additional uncertainty regarding study quality. However, the exclusion of grey literature may have resulted in the omission of studies with nonsignificant findings, which are generally less likely to be published (Rosenthal 1979). The bias assessment within the meta‐analysis suggested that publication bias in the parental overprotection group may have influenced the reported results. However, the nonsignificant publication bias assessment for other groups (i.e., secure attachment, avoidant attachment, anxious attachment, attachment quality and parental care) suggests minimal impact in these subgroups. Future reviews could consider broader inclusion by systematically incorporating both published and unpublished sources to enhance the comprehensiveness of the review.

Furthermore, the substantial degree of heterogeneity among the included studies, which encompassed variations in study design, attachment and suicide outcome measures, diverse conceptual frameworks underlying different attachment measures and cultural context, may impact the precision of the overall effect estimate. It is important to note that the statistical heterogeneity observed in the majority of subgroup meta‐analysis requires caution when interpreting the summary effects, including the possibility of variation in their direction. Although meta‐regressions were considered to explore potential sources of heterogeneity (e.g., sample characteristics (clinical vs. non‐clinical samples), study design, measurements, study quality or cultural context), they were not conducted due to the small number of studies in each subgroup. According to guidance, at least 10 studies are required to obtain reliable estimates of moderating effects in meta‐regression analyses (Higgins et al. 2019). Therefore, further research on the relationships between each attachment concepts and SI or SA is needed to identify sources of heterogeneity and to enable statistical testing of potential moderating effects.

### 5.5 Future Research

The relationship between avoidant or disorganised attachment and SI or SA remains unclear due to inconsistent findings and limited available studies. Adolescents with avoidant attachment may be less willing or able to report SI, affecting the reliability of self‐report data. Future research could address this by incorporating interviews with trusted clinical staff to create a supportive environment. Observer assessments may also help validate self‐reported disclosures. These methods could improve understanding of how avoidant attachment relates to SI. In addition, disorganised attachment is often overlooked, as it is typically viewed as a combination of avoidant and anxious attachment, marked by high levels of both (Mikulincer and Shaver 2010; Bartholomew and Horowitz 1991). However, disorganised attachment involves unique features such as fear, confusion and a lack of coherent strategies to manage distress that are not fully captured by these dimensions alone (Paetzold et al. 2015). Future studies should include specific measures of disorganised attachment to better understand its relationship with SI or SA.

To address heterogeneity caused by varied attachment and suicide measures, future research should adopt standardised or universally accepted tools. For attachment styles, tools such as the ECR (Brennan 1998) or RQ (Bartholomew and Horowitz 1991) could be used, both of which are widely regarded as reliable measures. For SI, the BSI (Beck and Steer 1991) is widely considered a valid measure, while observer assessments or medical records (Choi et al. 2022; Boudreaux et al. 2013) can be used to corroborate self‐reported SA. Consistency in measurement could strengthen the generation of more robust evidence on the relationship between attachment and SI or SA.

Although some longitudinal studies were included, most were cross sectional. Causal relationships cannot be definitively established, and reverse causality cannot be ruled out. Prospective longitudinal designs with long‐term follow‐up (e.g., well‐powered cohort studies) are needed to clarify directionality. In addition, most studies on mediators and moderators have used cross‐sectional designs. Longitudinal research is needed. Although several factors have been identified, they are often examined in isolation and without a comprehensive theoretical framework, limiting understanding of the underlying mechanisms. It would be beneficial to base research on comprehensive theoretical frameworks to better understand these factors. Adam's model of suicide explains how early attachment contributes to SI or SA (Adam 1994), identifying key mediators (e.g., self‐worth, emotion regulation and relationship skills) and moderators (e.g., negative life events). Applying this model could deepen understanding of the factors influencing the relationship between attachment and SI or SA in adolescents. This knowledge may inform clinical practice, leading to more effective interventions and better support, ultimately reducing suicide risk.

### 5.6 Clinical Implications

Evidence suggests that attachment is modifiable, with shifts between security and insecurity following changes in close relationship quality during adolescence and young adulthood (Booth‐LaForce et al. 2014; McConnell and Moss 2011; Pinquart et al. 2013). In light of this evidence, interventions targeting insecure attachment may help prevent SI and SA in adolescents (Diamond et al. 2010; Doolan and Bryant 2021; Levy et al. 2011). Interventions aimed at enhancing attachment security may contribute to suicide prevention. Since adolescents often live with attachment figures, systemic family influences shaping attachment may also affect youth development and suicidality (Morris‐Perez et al. 2024). Therefore, interventions delivered to and for parents to improve attachment security are important. These include programmes to increase parental sensitivity and availability during caregiving, improve caregiving quality, encourage parental involvement in suicide prevention and support parents in addressing their own issues to prevent negative effects on caregiving (Sutton 2019). Therapeutic support for adolescents to address negative caregiving experiences and unresolved parent–child relationship issues influencing attachment may also be beneficial (Van Ryzin et al. 2011). Given the importance of peer and school influences, including peer networks in interventions and developing school‐based programmes could further aid prevention (Morris‐Perez et al. 2024).

Several potential mediators (e.g., emotion problems and interpersonal factors) and moderators (e.g., social support and environmental sensitivity) were identified in this review. Therefore, interventions could focus on enhancing emotion regulation and interpersonal skills, fostering positive family and school environments and providing additional support within the community. To strengthen evidence for such programmes, future research should conduct RCT targeting specific mediators and moderators with sufficient sample sizes. These trials would help clarify the underlying causal mechanisms and identify key factors influencing the relationship between insecure attachment and SI or SA, thereby providing stronger evidence to enhance the effectiveness and practical implementation of these interventions.

## 6 Conclusion

This review provides evidence implicating insecure attachment to both SI and SA in adolescents. Emotional problems and interpersonal factors play a mediating role, while social support and environmental sensitivity serve as a moderating factor. These findings are consistent with established theoretical models of STB in adults (O'Connor and Kirtley 2018; Joiner 2005; Adam 1994), suggesting their applicability to adolescent populations. However, limitations such as measurement heterogeneity and the small number of studies on specific attachment concepts related to SI or SA warrant caution when interpreting these results. Overall, these findings highlight the potential importance of future interventions to enhance attachment security, improve emotional and interpersonal skills, support those with limited social networks and foster positive family and school environments in suicide prevention.

## Conflicts of Interest

The authors declare no conflicts of interest.

## Supporting information


**Appendix S1:** A full Boolean search string example.
**Appendix S2:** Data handling and comparability procedures.
**Appendix S3:** Details of measures of attachment and suicidal thoughts and behaviours.
**Appendix S4:** Quality assessment.
**Appendix S5:** Forest plots for the meta‐analysis of subgroups regarding suicidal ideation or suicide attempts.
**Appendix S6:** Funnel plots for each subgroup.
**Appendix S7:** Funnel plots after ‘Trim and Fill’ imputation (each subgroup).
**Appendix S8:** One study removed analysis.

## Data Availability

Data sharing not applicable to this article as no datasets were generated or analysed during the current study.
