# Supplementary material for: Attachment Concepts and Suicidal Thoughts and Behaviours in Adolescents: A Systematic Review and Meta‐Analysis
Source: Clin Psychol Psychother. 2026 Mar 17;33(2):e70251. doi: 10.1002/cpp.70251 (PMC12995856; doi:10.1002/cpp.70251)
Supplement: Supplementary file 1 — Appendix S1: A full Boolean search string example. Appendix S2: Data handling and comparability procedures. Appendix S3: Details of measures of attachment and suicidal thoughts and behaviours. Appendix S4: Quality assessment. Appendix S5: Forest plots for the meta‐analysis of subgroups regarding suicidal ideation or suicide attempts. Appendix S6: Funnel plots for each subgroup. Appendix S7: Funnel plots after ‘Trim and Fill’ imputation (each subgroup). Appendix S8: One study removed analysis. [file CPP-33-e70251-s001.docx]

# Supplementary file

## Appendix S1: A full Boolean search string example (Pubmed)

Search: **((attach* OR mother child relation* OR father child relation* OR peer relation* OR bond*) AND (suicid* OR self-injur* OR self-harm)) AND (child* OR teen* OR youth OR adolescen* OR young people OR young adult OR young person)**

("attach*"[All Fields] OR (("mother child"[Journal] OR ("mother"[All Fields] AND "child"[All Fields]) OR "mother child"[All Fields]) AND "relation*"[All Fields]) OR (("father s"[All Fields] OR "fathered"[All Fields] OR "fathers"[MeSH Terms] OR "fathers"[All Fields] OR "father"[All Fields] OR "fathering"[All Fields]) AND ("child"[MeSH Terms] OR "child"[All Fields] OR "children"[All Fields] OR "child s"[All Fields] OR "children s"[All Fields] OR "childrens"[All Fields] OR "childs"[All Fields]) AND "relation*"[All Fields]) OR ("peer"[All Fields] AND "relation*"[All Fields]) OR "bond*"[All Fields]) AND ("suicid*"[All Fields] OR "self injur*"[All Fields] OR ("self injurious behavior"[MeSH Terms] OR ("self injurious"[All Fields] AND "behavior"[All Fields]) OR "self injurious behavior"[All Fields] OR ("self"[All Fields] AND "harm"[All Fields]) OR "self harm"[All Fields])) AND ("child*"[All Fields] OR "teen*"[All Fields] OR ("adolescent"[MeSH Terms] OR "adolescent"[All Fields] OR "youth"[All Fields] OR "youths"[All Fields] OR "youth s"[All Fields]) OR "adolescen*"[All Fields] OR (("young"[All Fields] OR "youngs"[All Fields]) AND ("people s"[All Fields] OR "peopled"[All Fields] OR "peopling"[All Fields] OR "persons"[MeSH Terms] OR "persons"[All Fields] OR "people"[All Fields] OR "peoples"[All Fields])) OR ("young adult"[MeSH Terms] OR ("young"[All Fields] AND "adult"[All Fields]) OR "young adult"[All Fields]) OR (("young"[All Fields] OR "youngs"[All Fields]) AND ("person s"[All Fields] OR "personable"[All Fields] OR "personableness"[All Fields] OR "personal"[All Fields] OR "personalisation"[All Fields] OR "personalise"[All Fields] OR "personalised"[All Fields] OR "personalising"[All Fields] OR "personality"[MeSH Terms] OR "personality"[All Fields] OR "personalities"[All Fields] OR "personality s"[All Fields] OR "personalization"[All Fields] OR "personalize"[All Fields] OR "personalized"[All Fields] OR "personalizes"[All Fields] OR "personalizing"[All Fields] OR "personally"[All Fields] OR "personals"[All Fields] OR "persons"[MeSH Terms] OR "persons"[All Fields] OR "person"[All Fields])))

## Appendix S2: Data handling and comparability procedures

To ensure comparability across studies, control heterogeneity, and avoid the influence of non-independent data, the following strategies were employed:

(a) if studies measured attachment with mother and father separately, attachment with mother’s data were used;

(b) if studies measured SI with two different scale, data from the scale with a larger sample size was included;

(c) if studies measured both present SI and SI at the time of crisis, the data for present SI, which was measured most frequently among included studies, were included;

(d) if studies provided separate data for two independent groups, data from both groups was extracted;

(e) If studies analysed secondary data from the same original dataset, the effect size from the study with the largest sample size was used for the meta-analysis.

## Appendix S3: Details of measures of attachment and suicidal thoughts and behaviours

1. Attachment measures

| Abbreviation | Full name | Attachment concepts |
| --- | --- | --- |
| (1) security, avoidance, anxiety, and disorganisation | | |
| RQ | Relationship Questionnaire | secure, preoccupied, dismissing, fearful |
| ECR | Experiences in Close Relationships | anxious, avoidant |
| ECR-RS | Experiences in Close Relationships-Relationships Structure questionnaire | anxious, avoidant |
| CAI- | Child Attachment Interview | secure, preoccupied, dismissing, disorganised |
| ASS | Attachment Security Style Scale | secure |
| ASM | Attachment Style Measure | secure, anxious, avoidant |
| KSS | the Security Scale | secure |
| CAMIR-R | Questionnaire of Attachment Evaluation | security, family preoccupation |
| AAS | Adult Attachment Scale | secure, anxious, avoidant |
| (2) trust, communication, alienation, total score | | |
| IPPA | Inventory of Parent and Peer Attachment | trust, communication, alienation, total score |
| IPPA-6, IPPA-9, IPPA-12, IPPA-13, IPPA-15, IPPA-20 | Short version of IPPA:  6 items, 9 items, 12 items, 13 items, 15 items, 20 items of the original IPPA | trust, communication, alienation, total score |
| (3) care and overprotection | | |
| PBI | Parental Bonding Instrument | care, overprotection |
| Modified PBI | Short version of PBI | care, overprotection |
| (4) Others | | |
| FMAQ | Father/Mother Attachment Questionnaire | quality of emotional bond (QEB), separation anxiety and dependence (SAD), inhibition of exploration and individuality (IEI) |

2. Suicidal thoughts and behaviours measures

| Abbreviation | Full name |
| --- | --- |
| (1) Suicidal ideation (SI) | |
| C-SSRS | Columbia-Suicide Severity Rating Scale |
| SIQ | Suicide Ideation Questionnaire |
| SIQ-Jr | Suicidal Ideation Questionnaire-Junior |
| MFQ-SI (4/5) | Mood and Feelings Questionnaire (four or five items related to suicidal ideation) |
| SSI | Scale for Suicide Ideation |
| BSI | Beck Scale for Suicide Ideation |
| PANSI | Positive and Negative Suicide Ideation inventory |
| SIS | Suicide Intent Scale |
| C-SSRS (ISI) | Intensity of Ideation subscale of the Columbia-Suicide Severity Rating Scale |
| SSOSI | Self-rating scale of suicidal ideation |
| (2) Suicide attempts (SA) | |
| PSS | Paykel Suicide Scale |
| SBS | Suicidal Behaviours Schedule (Cédula de Conductas Suicidas) |
| SHF | Columbia University Suicide History Form |

Noted: The attachment measurements across the included studies varied substantially, with 26 different measures. Fifty-two studies utilised self-report measures and two studies utilised interviews. The measures assessed the following domains: (1) security, avoidance, anxiety, and disorganisation; (2) trust, communication, alienation, and total quality; and (3) care and overprotection. Attachment research focuses primarily on parents, caregivers, and family, and a small number of studies assessed peer attachment. Concerning suicidal thoughts and behaviours, 34 studies examined suicidal ideation alone, 12 studies suicide attempts alone, and eight studies examined both and separated them into two categories. In total, 18 distinct measures were used to evaluate suicidal ideation. The single-item question, such as “Have you ever thought about killing yourself? or “Have you ever tried to kill yourself?” (n=25) was the most prevalent. Twenty-six studies utilised self-report questionnaires, one utilised medical records to identify participants with a history of suicide attempt, and two used interview-based assessment.

## Appendix S4: Quality assessment

|  | Q1 | Q2 | Q3 | Q4 | Q5 | Q6 | Q7 | Q8 | Q9 | Q10 | Q11 | Q12 | Q13 | Q14 | Q15 |
| --- | --- | --- | --- | --- | --- | --- | --- | --- | --- | --- | --- | --- | --- | --- | --- |
| Author (published Year) | Research Question | Study Population | Participation Rate of Eligible Persons | Recruitment | Sample Size Justification | Exposure prior Outcome | Sufficient Timeframe | Exposure Levels | Exposure Measures | Repeated Exposure Assessment | Outcome Measures | Blinding of Outcome Assessors | Follow-up Rate (>80%) | Statistical Analyses | Overall Quality Rating |
| Bakken et al. (2025) | YES | YES | YES | YES | NR | YES | YES | NA | YES | NA | YES | NA | YES | YES | GOOD |
| Dong et al. (2024) | YES | YES | YES | YES | NR | YES | YES | NA | YES | YES | YES | NA | YES | YES | GOOD |
| Shin and Bae (2024) | YES | YES | YES | YES | NR | YES | YES | NA | YES | YES | NO | NA | YES | YES | GOOD |
| Bakken et al. (2024) | YES | YES | YES | YES | NR | YES | YES | NA | YES | NA | YES | NA | NO | YES | FAIR |
| Yang et al. (2023) | YES | YES | YES | YES | NR | NA | NA | NA | YES | NA | YES | NA | NA | YES | GOOD |
| Myerson et al. (2023) | YES | YES | NR | YES | NR | NA | NA | NA | YES | NA | NO | NA | NA | YES | GOOD |
| Cohen and Stutts (2023) | YES | YES | YES | YES | NR | NA | NA | NA | YES | NA | NO | NA | NA | YES | GOOD |
| Guo et al. (2023) | YES | YES | YES | YES | NR | NA | NA | YES | YES | NA | YES | NA | YES | YES | GOOD |
| Novak et al. (2023) | YES | YES | YES | YES | NR | NA | NA | NA | NO | NA | NO | NA | NA | YES | FAIR |
| Fattouh et al. (2022) | YES | YES | YES | YES | YES | NA | NA | YES | NO | NA | YES | NA | NA | YES | FAIR |
| Ding et al. (2022) | YES | YES | NR | YES | NR | NA | NA | NA | NR | NA | NO | NA | NA | YES | FAIR |
| Guo et al. (2021) | YES | YES | YES | YES | NR | NA | NA | NA | YES | NA | YES | NA | NA | YES | GOOD |
| Hunt et al. (2021) | YES | YES | YES | YES | NR | NA | NA | NA | YES | NA | YES | NA | YES | YES | GOOD |
| Mirkovic et al. (2021) | YES | YES | YES | YES | NR | NA | NA | NA | YES | NA | NO | NA | NA | YES | GOOD |
| Herres et al. (2021) | YES | YES | YES | YES | NR | NA | NA | NA | YES | NA | YES | NA | YES | YES | GOOD |
| Hermosillo-De-La-Torre et al. (2021) | YES | YES | NR | YES | NR | NA | NA | NA | YES | NA | YES | NA | NA | YES | GOOD |
| Waraan et al. (2021) | YES | YES | YES | YES | NR | NA | NA | NA | NR | NA | NR | NA | NA | YES | FAIR |
| Potard et al. (2020) | YES | YES | YES | YES | NR | NA | NA | NA | YES | NA | YES | NA | NA | YES | GOOD |
| Cantón-Cortés et al. (2020) | YES | YES | YES | YES | NR | NA | NA | NA | NR | NA | NR | NA | NA | YES | FAIR |
| Moyano et al. (2020) | YES | YES | YES | YES | YES | NA | NA | NA | YES | NA | NO | NA | NA | YES | GOOD |
| Chang et al. (2019) | YES | YES | YES | YES | NR | NA | NA | NA | YES | NA | YES | NA | YES | YES | GOOD |
| Handley et al. (2018) | YES | YES | NR | YES | NR | NA | NA | NA | YES | NA | NO | NA | NA | YES | GOOD |
| Cerutti et al. (2018) | YES | YES | NR | YES | NR | NA | NA | NA | YES | NA | NO | NA | NA | YES | GOOD |
| Bar-Zomer and Klomek (2018) | YES | YES | YES | YES | NR | NA | NA | NA | YES | NA | YES | NA | NA | YES | GOOD |
| Ibrahim et al. (2018) | YES | YES | YES | YES | NR | NA | NA | NA | YES | NA | YES | NA | YES | YES | GOOD |
| Sharif and Akhtar (2018) | YES | YES | YES | NO | NR | NA | NA | NA | YES | NA | YES | NA | NA | NO | POOR |
| Nunes and Mota (2016) | YES | YES | NR | YES | NR | NA | NA | NA | YES | NA | YES | NA | NA | YES | GOOD |
| Zisk et al. (2016) | YES | YES | YES | YES | NR | NA | NA | NA | YES | NA | YES | NA | YES | YES | GOOD |
| Sharaf et al. (2016) | YES | YES | YES | YES | YES | NA | NA | NA | NO | NA | YES | NA | NA | YES | FAIR |
| Lee (2016) | YES | YES | NR | YES | NR | NA | NA | NA | Partially Yes (PBI scale) | NA | NO | NA | NA | YES | GOOD |
| Li et al. (2016) | YES | YES | NR | YES | NR | NA | NA | NA | YES | NA | NO | NA | NA | YES | GOOD |
| Sheftall et al. (2014) | YES | YES | YES | YES | NR | NA | NA | NA | YES | NA | YES | NA | NA | YES | GOOD |
| Saffer et al. (2014) | YES | YES | NR | YES | NR | NA | NA | NA | YES | NA | NO | NA | NA | YES | GOOD |
| Cruz et al. (2014) | YES | YES | NR | YES | NR | NA | NA | NA | YES | NA | NO | NA | NA | YES | GOOD |
| Venta et al. (2014) | YES | YES | NR | YES | NR | NA | NA | NA | YES | NA | NO | NA | NA | YES | GOOD |
| Venta and Sharp (2014) | YES | YES | YES | YES | NR | NA | NA | NA | YES | NA | NO | NA | NA | YES | GOOD |
| Maršanić et al. (2013) | YES | YES | YES | YES | NR | NA | NA | NA | NO | NA | NO | NA | NA | YES | FAIR |
| Sheftall et al. (2013) | YES | YES | YES | YES | NR | NA | NA | NA | YES | NA | YES | NA | NA | NO | FAIR |
| Phuong et al. (2013) | YES | YES | YES | YES | NR | NA | NA | NA | NO | NA | NO | NA | NA | YES | FAIR |
| Peltzer and Pengpid (2012) | YES | YES | YES | YES | NR | NA | NA | YES | NO | NA | NO | NA | NA | YES | FAIR |
| Maimon et al. (2010) | YES | YES | NR | YES | NR | YES | YES | NA | NO | NA | NO | NA | YES | YES | FAIR |
| Maimon and Kuhl (2008) | YES | YES | YES | YES | NR | YES | YES | NA | NO | NA | NO | NA | NA | YES | FAIR |
| Peter et al. (2008) | YES | YES | YES | YES | YES | NA | NA | NA | NO | NA | NO | NA | NA | YES | FAIR |
| Kidd and Shahar (2008) | YES | YES | NR | YES | NR | NA | NA | NA | YES | NA | YES | NA | NA | YES | GOOD |
| Nrugham et al. (2007) | YES | YES | YES | YES | NR | YES | YES | NA | YES | NA | NO | NA | YES | YES | GOOD |
| Silviken and Kvernmo (2006) | YES | YES | YES | YES | NR | NA | NA | NA | NO | NA | NO | NA | NA | YES | FAIR |
| Lai and McBride‐Chang (2001) | YES | YES | NR | YES | NR | NA | NA | NA | NO | NA | NO | NA | NA | NO | POOR |
| DiFilippo and Overholser (2000) | YES | YES | YES | YES | NR | NA | NA | NA | YES | NA | YES | NA | NA | YES | GOOD |
| Fergusson et al. (2000) | YES | YES | NR | YES | NR | YES | YES | NA | YES | NA | NO | NA | YES | YES | GOOD |
| Lessard and Moretti (1998) | YES | YES | YES | YES | NR | NA | NA | NA | NO | NA | NO | NA | NA | NO | POOR |
| Beautrais et al. (1996) | YES | YES | YES | YES | NR | NA | NA | NA | YES | NA | YES | NA | NA | YES | GOOD |
| Adam et al. (1994) | YES | YES | NR | YES | NR | NA | NA | NA | YES | NA | YES | NA | NA | YES | GOOD |
| Martin and Waite (1994) | YES | YES | YES | YES | NR | NA | NA | NA | YES | NA | NO | NA | NA | NO | FAIR |
| Strang and Orlofsky (1990) | YES | YES | NR | YES | NR | NA | NA | NA | YES | NA | YES | NA | NA | NO | FAIR |

Note: *CD, cannot determine; NA, not applicable; NR, not reported.

Detailed information used in rating each question and the overall quality: A ‘no’ may apply to each question if, for example, the research question is not clearly defined, the sample is not representative of the target population, the recruitment strategy is not sufficiently clear to allow replication based on the description, measures that are not psychometrically validated, the use of an unvalidated translated version of a psychometrically validated measure, a longitudinal design is used but shows high attrition, confounders are not controlled for in the design or analysis, or the analysis fails to address the research question. Responses of ‘cannot determine’ and ‘not reported’ are noted as potential flaws in the quality assessment. For instance, if ‘cannot determine’ or ‘not reported’ is chosen regarding sample size justification, this is considered a minimal risk of bias and is unlikely to significantly affect the results. However, if the same response is selected for the evaluation of measures, it can indicate a more substantial concern. For example, if the sample population's first language is not English and the measures section does not specify whether a translated version was used or if that translated version has been validated or shown high reliability in previous research, this lack of clarity may introduce bias and could potentially influence the results. In such cases, it can be interpreted as a ‘no’ for this question. Questions marked as ‘not applicable’ were excluded from consideration when determining the overall quality rating. A 'good' study is characterised by minimal risk of bias (no ‘no’ responses selected), indicating that its results are reliable. In contrast, a 'fair' study may exhibit some bias (one ‘no’ response selected), but it is not significant enough to invalidate its findings. A ‘poor’ rating suggests a substantial risk of bias (two or more ‘no’ responses selected), which can influence the results, meaning that the findings should be interpreted with caution. For the evaluation of the measurement method, if ‘no’ or ‘not reported’ responses appear in both the measurement of suicidal ideation and attachment, they were combined into a single ‘no’ or ‘not reported’.

Detailed explanation for ‘fair’ and ‘poor’ studies:

‘Fair’ studies: Regarding the 17 'fair' studies, one was rated as 'fair' due to a low follow-up rate (less than 60%). Seven included studies (76-82) suggested confounding factors (e.g., age, gender, negative life events, or mental health problems) may affect the relationship between attachment and suicidal ideation or attempts. Consequently, three studies received a 'fair' rating because they did not account for potential confounders. The other 13 studies received a “fair” rating because they did not utilise validated attachment measures. Specifically, nine of these studies utilised an unvalidated translated version of psychometrically validated measures that have not been demonstrated to have validity or reliability in prior research, nor have they undergone standard validation testing. The other four studies employed scales, which included items like "I know my family will always be there for me" or " How close do you feel to your mother/father”. While these items may reflect some aspects of the emotional bonds between adolescents and their parents or family, which are central to attachment, they do not fully capture the complexity of this construct. Attachment encompasses not only emotional bonds but also the emotional and attachment needs that arise during times of need, leading to behaviours in moments of distress, such as seeking support or comfort from attachment figures. Consequently, these measures may overlook critical aspects of attachment, limiting validity and confidence in results. In addition, single-item measures of suicidal ideation or suicide attempts may also impact the accuracy and reliability of evaluations of suicidal ideation or suicide attempts. However, reviews (Batterham et al., 2015; Carter et al., 2017) have indicated that there is no gold-standard measurement for suicidal ideation or suicide attempts. Some studies (Ammerman & Law, 2022; Millner et al., 2015) have shown that single-item measures of suicidal ideation or suicide attempts can provide reasonably accurate detection. Therefore, the use of single-item measures is considered acceptable in this review.

‘Poor’ studies: Two studies (J.c & M.m, 1998; Lai & McBride-Chang, 2001) failed to account for confounding factors and used unvalidated translated or interview-based measures. One study (Sharif & Akhtar, 2018) lacked detail in recruitment and selection procedures and overlooked potential confounders.

References:

Ammerman, B. A., & Law, K. C. (2022). Using intensive time sampling methods to capture daily suicidal ideation: A systematic review. Journal of Affective Disorders, 299, 108–117.

Batterham, P. J., Ftanou, M., Pirkis, J., Brewer, J. L., Mackinnon, A. J., Beautrais, A., Fairweather-Schmidt, A. K., & Christensen, H. (2015). A systematic review and evaluation of measures for suicidal ideation and behaviors in population-based research. Psychological Assessment, 27(2), 501.

Cantón-Cortés, D., Cortés, M. R., & Cantón, J. (2020). Child sexual abuse and suicidal ideation: The differential role of attachment and emotional security in the family system. International Journal of Environmental Research and Public Health, 17(9), 3163.

Carter, G., Milner, A., McGill, K., Pirkis, J., Kapur, N., & Spittal, M. J. (2017). Predicting suicidal behaviours using clinical instruments: Systematic review and meta-analysis of positive predictive values for risk scales. The British Journal of Psychiatry, 210(6), 387–395.

DiFilippo, J. M., & Overholser, J. C. (2000). Suicidal ideation in adolescent psychiatric inpatients as associated with depression and attachment relationships. Journal of Clinical Child Psychology, 29(2), 155–166.

Fergusson, D. M., Woodward, L. J., & Horwood, L. J. (2000). Risk factors and life processes associated with the onset of suicidal behaviour during adolescence and early adulthood. Psychological Medicine, 30(1), 23–39. https://doi.org/10.1017/S003329179900135X

J.c, L., & M.m, M. (1998). Suicidal ideation in an adolescent clinical sample: Attachment patterns and clinical implications. Journal of Adolescence, 21(4).

Lai, K. W., & McBride-Chang, C. (2001). Suicidal ideation, parenting style, and family climate among Hong Kong adolescents. International Journal of Psychology, 36(2), 81–87.

Millner, A. J., Lee, M. D., & Nock, M. K. (2015). Single-item measurement of suicidal behaviors: Validity and consequences of misclassification. PloS One, 10(10), e0141606.

Phuong, T. B., Huong, N. T., Tien, T. Q., Chi, H. K., & Dunne, M. P. (2013). Factors associated with health risk behavior among school children in urban Vietnam. Global Health Action, 6, 1–9. https://doi.org/10.3402/gha.v6i0.18876

Sharaf, A. Y., Thompson, E. A., & El-Salam, H. F. A. (2016). Perception of parental bonds and suicide intent among Egyptian adolescents. Journal of Child and Adolescent Psychiatric Nursing, 29(1), 15–22.

Sharif, S., & Akhtar, T. (2018). Relationship of late adolescent’s attachment styles with suicidal ideation and resilience. Foundation University Journal of Psychology, 2(2), 96–120.

Sheftall, A. H., Schoppe-Sullivan, S. J., & Bridge, J. A. (2014). Insecure Attachment and Suicidal Behavior in Adolescents. Crisis, 35(6), 426–430. https://doi.org/10.1027/0227-5910/a000273

Waraan, L., Mehlum, L., Rognli, E. W., Czajkowski, N. O., & Aalberg, M. (2021). Associations between insecure attachment styles to parents and suicidal ideation in adolescents with depression. Scandinavian Journal of Child and Adolescent Psychiatry and Psychology, 41–51.

## Appendix S5: Forest plots for the meta-analysis of subgroups regarding suicidal ideation or suicide attempts

1. Forest plot for the relationship between secure attachment and suicidal ideation

2. Forest plot for the relationship between avoidant attachment and suicidal ideation

3. Forest plot for the relationship between anxious attachment and suicidal ideation

4. Forest plot for the relationship between attachment quality and suicidal ideation

5. Forest plot for the relationship between parental care and suicidal ideation

6. Forest plot for the relationship between parental overprotection and suicidal ideation

7. Forest plot for the relationship between parental care and suicide attempts

## Appendix S6: Funnel plots for each subgroup

1. Funnel plot for the relationship between secure attachment and suicidal ideation

2. Funnel plot for the relationship between avoidant attachment and suicidal ideation

3. Funnel plot for the relationship between anxious attachment and suicidal ideation

4. Funnel plot for the relationship between attachment quality and suicidal ideation

5. Funnel plot for the relationship between parental care and suicidal ideation

6. Funnel plot for the relationship between parental overprotection and suicidal ideation

7. Funnel plot for the relationship between parental care and suicide attempts

## Appendix S7: Funnel plots after ‘Trim and Fill’ imputation (each subgroup)

1. Funnel plot after ‘trim and fill’ imputation for the relationship between secure attachment and suicidal ideation

2. Funnel plot after ‘trim and fill’ imputation for the relationship between avoidant attachment and suicidal ideation

3. Funnel plot after ‘trim and fill’ imputation for the relationship between anxious attachment and suicidal ideation

4. Funnel plot after ‘trim and fill’ imputation for the relationship between attachment quality and suicidal ideation

5. Funnel plot after ‘trim and fill’ imputation for the relationship between parental care and suicidal ideation

6. Funnel plot after ‘trim and fill’ imputation for the relationship between parental overprotection and suicidal ideation

7. Funnel plot after ‘trim and fill’ imputation for the relationship between parental care and suicide attempts

## Appendix S8: One study removed analysis

1. One study removed analysis for the relationship between secure attachment and suicidal ideation

2. One study removed analysis for the relationship between avoidant attachment and suicidal ideation

3. One study removed analysis for the relationship between anxious attachment and suicidal ideation

4. One study removed analysis for the relationship between attachment quality and suicidal ideation

 5. One study removed analysis for the relationship between parental care and suicidal ideation

6. One study removed analysis for the relationship between parental overprotection and suicidal ideation

7. One study removed analysis for the relationship between parental care and suicide attempts
